# Energy-based generative models for monoclonal antibodies

**DOI:** 10.1080/19420862.2025.2584935

**Published:** 2025-11-25

**Authors:** Paul Pereira, Hervé Minoux, Aleksandra M. Walczak, Thierry Mora

**Affiliations:** a Laboratoire de Physique de l’École Normale supérieure, CNRS, PSL University, Sorbonne Université, and Université de Paris, Paris, France; b Sanofi, Vitry-Sur-Seine, France

**Keywords:** Computational design, AI models, multi-objective optimization

## Abstract

Since the approval of the first antibody drug in 1986, a total of 162 antibodies have been approved for a wide range of therapeutic areas, including cancer, autoimmune, infectious, or cardiovascular diseases. Despite advances in biotechnology that accelerated the development of antibody drugs, the drug discovery process for this modality remains lengthy and costly, requiring multiple rounds of optimizations before a drug candidate can progress to preclinical and clinical trials. This multi-optimization problem involves increasing the affinity of the antibody to the target antigen while refining additional biophysical properties that are essential to drug development such as solubility, thermostability or aggregation propensity. Additionally, antibodies that resemble natural human antibodies are particularly desirable, as they are likely to offer improved profiles in terms of safety, efficacy, and reduced immunogenicity, further supporting their therapeutic potential. In this article, we explore the use of energy-based generative models to optimize a candidate monoclonal antibody. We identify tradeoffs when optimizing for multiple properties, focusing on solubility, humanness and affinity and use the generative model we develop to generate candidate antibodies that lie on optimal Pareto fronts with respect to these properties.

## Introduction

Monoclonal antibodies are an important type of biological drug, with 162 therapeutic antibodies currently approved to be used to treat a variety of diseases for different therapeutic areas such as cancer or inflammation. Their development, however, is costly, time consuming and prone to failure. Antibodies are Y-shaped proteins that specifically bind to an antigen. The number of possible antibodies that can be designed is too large to search exhaustively when trying to find an antibody that will bind sufficiently strongly to a target antigen. In addition to affinity, there are a number of other desirable properties that the candidate antibody must have in order to function properly as a drug. For example, it is desirable for the antibody not to have exposed hydrophobic patches that could cause aggregation issues and to be soluble. The antigenicity of the candidate antibody must also be as low as possible in order to avoid an immune reaction in the patient. These constraints further reduce the number of drug candidates and must be taken into account as early as possible in the development process.

We focus on the development of energy-based generative models for monoclonal antibodies. Our work falls within a broad range of methods utilizing wet-lab experiments and machine learning models to optimize antibodies for drug development. In-vitro assays can be used to characterize antibodies and filter-out ones with poor affinity to the target antigen or poor developability properties.^1^ The development of high-throughput assays has made it possible to characterize large numbers of sequences in batches. Methods like phage or yeast display^2,3^ can be used to identify a few antibodies that bind to a target antigen out of a large library of diverse antibodies or to optimize such candidates by comparing many different mutants of the same wild-type sequence.^4^ However, high-throughput methods tend to provide less accurate information than low-throughput methods such as surface plasmon resonance^5^ and can still take a long time to run compared to in-silico methods.

Machine learning models can be trained on the data generated by these wet-lab experiments.^6^ These models can then be used to evaluate the properties of large libraries of antibodies in order to filter-out poor drug candidates and reduce the list of candidates to be tested using wet-lab experiments. This process may lower the experimental cost, allowing for the use of lower-throughput methods when possible. For example, Ref.^7^ used affinity information about $1\times 10^{4}$ variants of the clinical antibody trastuzumab binding to Her2 to train a machine learning model that was then used to predict the affinity of $1\times 10^{8}$ trastuzumab variants and to identify top binders. The top predicted binders were further tested using in-silico models for viscosity, clearance, solubility and immunogenicity to end up with a few thousand highly optimized antibodies. While this approach has demonstrated its efficacy, its success relies on optimal candidates being already present in the library of candidates. This is not guaranteed and depends on the way the library is designed.

Alternatively, generative algorithms directly generate optimized candidate antibodies. Biswas et al.^8^ successfully combined a predictive model for the fluorescence of green fluorescent proteins and energy-based sampling methods to generate new amino sequences of GFP from Aequorea victoria with increased fluorescence. Jain et al.^9^ demonstrated the ability of autoregressive models to learn to generate amino acid sequences optimized with respect to the output of a proxy predictive model for three different tasks (anti-microbial peptide, TFBind 8 and GFP). More recently, Bennet et al.^10^ demonstrated the ability of their structure generative method to design de-novo single-domain VHH that bind to target antigens for which the epitope is specified.

We develop and analyze a method for single-round optimization of monoclonal antibodies. We start from a wild type (WT) sequence of the variable part of an antibody that binds to a target antigen. While this WT antibody is functional (i.e. binds to the target antigen), we want to improve some of its properties: further increase its affinity to the target antigen or reduce the risk of aggregation. Furthermore, we want to improve these properties by performing a small number of mutations, in order to prevent the loss of functionality from the WT antibody. Finally, in order to decrease the antigenicity of the antibody, we aim to generate antibodies that are as human-like as possible. In short, we explore the tradeoffs generative models need to consider to produce candidate antibodies that satisfy properties needed in practical antibody discovery: solubility, lack of antigenicity and strong affinity.

### Results

#### Multiple objective optimization

Each antibody is made of two copies of a heterodimer, each composed of a heavy chain and a light chain. The variable regions of the antibodies contain the complementarity-determining regions (CDR), flexible loop structures that make up a large part of the paratopes (the section of the antibody that is in contact with the antigen). The heavy chain and light chain each contain three CDR regions: CDRH1, CDRH2 and CDRH3 for the heavy chain and CDRL1, CDRL2 and CDRL3 for the light chain. In this work, we will restrict the optimization to the heavy chain sequence for simplicity although our method can easily be modified to include the light chain.

We use a multi-objective optimization approach. We consider a set of biophysical properties $f_{p}(x)$, assumed to be a function of the antibody sequence x, which we wish to maximize all at once. We expect trade-offs to emerge since optimizing some properties may come at the cost of others. We look for Pareto optimal solutions, defined as sequences which cannot be improved in any property without being degraded in another one. Combinations of biophysical properties achieved by Pareto optimal sequences form the Pareto front.

We do not have access to the functions $f_{p}$ directly for arbitrary sequences x. Instead, we will exploit predictive machine learning models ${\hat{f}}_{p}$ trained on large datasets to generate sequences that are close to the Pareto front. We are interested in the single-round setting: we assume that once our generative method has selected candidates for us to test, we will be able to validate those candidates using wet-lab experiments only once, with a fixed budget of B sequences to be tested. Since we do not expect the models ${\hat{f}}_{p}$ to perfectly reproduce the desired properties, we wish to generate a diverse set of B candidates to maximize the probability that at least one of them will pass validation. We further require that these sequences be different from the ones included in the datasets used to train the models.

In the following, we will focus on two main biophysical properties: solubility (sol) and affinity (aff). Our generative process incorporates the humanness of sequences through the distribution of natural antibodies $p_{HUM}(x)$, as given by an auto-regressive transformer previously trained on 558 million human heavy chain sequences from the OAS database.^11,12^ We define p(x) for our generative model to be as close as possible to $p_{HUM}$ (as measured by the Kullback-Leibler divergence) while maximizing the mean values of $f_{sol}$ and $f_{aff}$. This gives us the explicit expression:

(1) $$p(x)=\frac{1}{Z}p_{HUM}(x)e^{-E(x)/T}$$

(2) $$E(x)=-w{\hat{f}}_{a\mathrm{ff}}(x)-(1-w){\hat{f}}_{s\mathrm{ol}}(x),$$

where Z is a normalization, T may be interpreted as a temperature controlling to cloneness to the Pareto front, and 0≤w≤1 is a weight controlling the importance of affinity versus solubility in the optimization.

**Figure 1.**
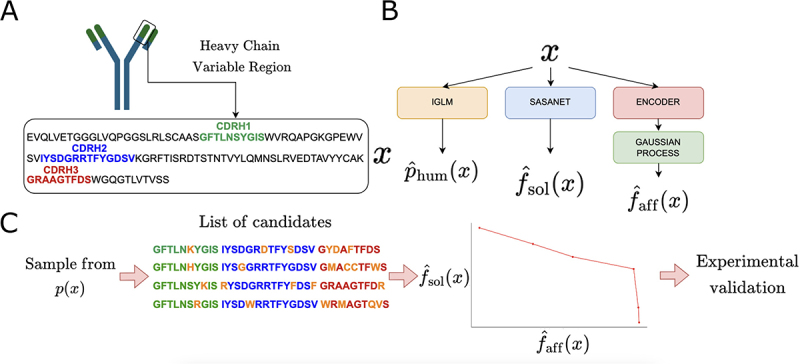
Overview of the generative process. A. Our generative model generates the heavy chain sequence of an antibody undergoing lead optimization. The goal is to find a small number of mutations in the CDRs to improve affinity and solubility. B. For humaness, our predictive model is IGLM, an autoregressive transformer which outputs a log probability estimate of the input heavy chain being human. For solubility, we use a convolutional neural network that estimates the per-residue sasa and combines these estimates with hydrophobicity weights. For the affinity we use a Gaussian process in combination with a protein language model encoder. C Energy-based sampling is used to generate mutants of the wild-type sequence with up to $d_{lim}$ mutations. The closest sequences to the pareto front are selected for wet-lab validation.

We then use two energy-based generative methods to sample new heavy chain sequences from p(x) (Figure 1): Metropolis Hastings (MCMC) and the amortized Monte Carlo method GFlowNet.^13,14^ Markov Chain Monte Carlo (MCMC) is a method with theoretical guarantees of convergence, allowing exact sampling from the target distributions. It provides a valid baseline to compare with GFlowNet, a novel deep learning method with the potential to generate sequences faster than MCMC. GFlowNet has been shown empirically to generate better and more diverse amino acid sequence candidates than other generative methods, for example, on the task of generating new anti-microbial peptides.^9^ Details of these procedures are given in the Methods section.

Below, we apply this approach to generate optimized binders to the HR2 region of the SARS-CoV-2 spike protein peptide (CB-119) whose amino acid sequence is PDVDLGDISGINAS.

#### Affinity model

Antibody-antigen affinity is often defined in terms of the dissociation constant ($K_{d}$) between an antibody and its antigen. High affinity means low $K_{d}$, so we define $f_{aff}=\log(1\;nM/K_{d})$. In order to train our affinity prediction model, we use the dataset from Engelhart et al.^15^ A library of variants was built by first identifying a pair of heavy and light chains that produce an antibody (Ab-14) that binds to CB-119. The heavy chain of Ab-14 has 33 CDR amino acids that were modified to generate 22,000 mutants: 594 single mutants were generated by doing a saturated deep mutational scan over the CDRH1, CDRH2 and CDRH3. In addition, 3,671 double mutants and 22,188 triple mutants were generated by performing random substitutions in the CDRs. The affinity of each sequence was measured 3 times using the AlphaSeq assay to compute their $K_{d}$ with respect to the CB-119 SARS-CoV-2 peptide. Of the 26,453 sequences, only 13,921 sequences had affinity measurements. We approximate their true $K_{d}$ as the average over replicates and remove the sequences for which there are no measurements.

For our affinity prediction method, we use a Gaussian Process. The Gaussian process assumes that outputs (affinities) are drawn from a multivariate Gaussian distribution whose covariance between two outputs depends on the distance between their input sequences x, and uses Gaussian integration rules to predict the posterior for the affinity of any sequence x as a function of the training data:

(3) $$f_{aff}(x)∼N(\mu (x;D),\sigma^{2}(x;D)),$$

where $D=(x_{i},f_{aff}(x_{i}){)}_{i}$ is the training dataset.

We define multiple models using different embeddings for the amino acid sequences and use a Radial Basis Function (RBF) as our kernel function (see Methods).

The parameters of the kernel function (δ and λ) are fitted by minimizing the marginal likelihood loss function (see Eq. 14) for 600 steps using a learning rate of $10^{-3}$ (see Methods for details). We utilize the Gpytorch^16^ framework to implement the Gaussian Process and fit the parameters.

To score sequences, we define four different versions of the affinity function to be used in the multiple-objective optimization: ${\hat{f}}_{a\mathrm{ff}}(x;\beta )=\mu (x)+\beta \sigma (x)$, with β=−1,0,1,2. When β is positive we get back the Upper Confidence Bound (UCB) acquisition function^17^ which gives priority to sequences with high uncertainty and encourages exploration of the sequence space. When β is negative, we obtain the Lower Confidence Bound (LCB) acquisition function, a pessimistic estimate of the affinity of the sequence. Using LCB encourages the model to generate sequences close to the ones in the training set.

**Figure 2.**
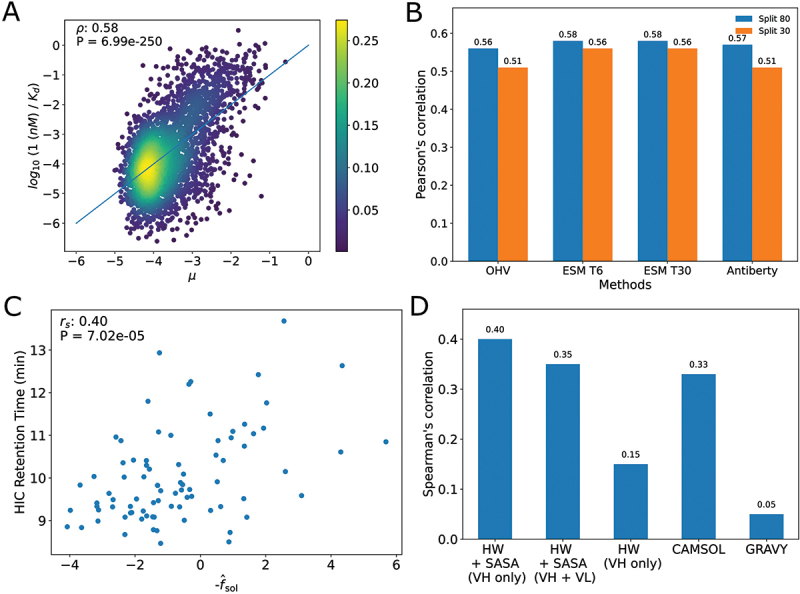
A. Density plot showing results from predicting kd using Gaussian process B. Pearson’s correlation coefficient for different choices of embedding and different training set sizes C. Scatter plot comparing hic rt to solubility score for 83 monoclonal antibodies (not shown are two outliers with HIC RT equal to 25). The Spearman correlation coefficient and p-value are computed with the outliers. D. Spearman’s correlation score for different solubility methods. HW + SASA refers to the method we retain for the generative process, HW is similar to HW + SASA except the predicted sasa for each residue is set to 1. Camsol refers to the Camsol method^18^ and gravy refers to the biopython hydrophobicity score function.

We split the dataset into a training and a validation set. We refer to the training set as the set of sequences that are used to fit the parameters of the kernel function and to make a prediction. In order to test the ability of the Gaussian process to correctly predict the affinity to CB-119, we first built a training set of 80% of D and validate the model on the remaining 20%. The Pearson correlation score between the log of the measured association constant $f_{aff}$ and the predicted affinity $\mu_{aff}$ on the validation set for the best choice of embedding and when training on 80% of the data is 0.58 (Figure 2A) showing that the model is able to discriminate between high- and low-affinity sequences.

Using the protein language model (PLM) ESM2^19^ for the embeddings gives a modest improvement over the simpler one-hot vector encoding of the sequences (Figure 2B). We observe no differences between using a smaller version of ESM (ESM-T6) versus a larger network (ESM-T30). Using an antibody-specific protein, language model (Antiberty)^20^ gives worse results than a more general PLM (Figure 2). We asked whether reducing the amount of training data significantly decreases the performance of the model. When we reduce the size of the training set to only 30% of the data, the Pearson correlation coefficient decreases by only 0.02 when using the ESM-T6 PLM to compute the embedding. Since the running time for the prediction of a Gaussian Process is quadratic in the number of sequences in the training set, evaluating new sequences during the generative processes, we use the ESM-T6 embeddings and train the Gaussian Process on 30% of the dataset to limit the computational cost. In addition, we also considered adding out-of distribution-negative examples in order to try to improve the quality of the model. To do this, we generated new mutants by replacing the CDRs of the WT with randomly generated sequences. The CDRs were generated by drawing uniformly over the set of amino acids for each of 33 possible positions. The assumption we use is that by randomizing the CDRs it is very likely that the sequences we generate are far away (in terms of hamming distance) from the WT and are therefore likely to be non-binders. We therefore assign to each sequence a large $K_{a}=10^{7}M^{-1}$. We generated 14,000 such negative examples and retrained a separate model using the ESM-T6 embeddings where the training dataset contains the original 80% training split and the generated negative examples. We validated the performance of the model on the original 20% training split and found a drop in performance from 0.58 to 0.55 pearson correlation (see SI Fig S15) compared to not using the negative data. Therefore, we decided to use the model not trained on the negative examples for the rest of the paper.

#### Solubility model

The solubility of a monoclonal antibody is related to different factors that can impact the efficacy of the drug. Antibodies can aggregate when stored in an aqueous solution which will result in a painful reaction when the drug is administered to the patient. In addition, aggregates can become a target for the immune system and increase the antigenicity of the drug. Hydrophobic interaction chromatography (HIC) estimates the solubility of antibodies by measuring the time it takes the antibody to cross a buffer. Stronger hydrophobic amino acids result in longer times.

We use a method developed by Jain et al.^21^ to estimate the solubility of an antibody based on its sequence. Given a sequence of amino acid of length L: $x=x_{1},x_{2},\ldots ,x_{L}$, the solubility score of the sequence is defined as

(4) $${\hat{f}}_{s\mathrm{ol}}(x)=-\sum_{j=1}^{L}S\mathrm{ASA}(j,x_{j})HW(x_{j})+\mathrm{const},$$

where $S\mathrm{ASA}(i,S_{i})$ is the residue solvent-accessible surface area of amino acid $a_{i}$ at position i, and $HW(x_{i})$ is one of the 20 hydrophobic weights describing the hydrophobicity of the amino acid $x_{i}$. We use the solubility weights provided in Jain et al.^21^ and train a convolutional neural network to predict the per-residue SASA score of each amino acid in an antibody heavy chain sequence.

Since the per-residue SASA score is not available from the sequence alone, Jain et al. compute the per-residue SASA for 902 antibody structures identified from the RCSB PDB database using the Shrake-Rupley algorithm^22^ and train a random forest regressor to predict the per-residue SASA of residues in the variable region from the sequence alone. They then use a private dataset of 5000 antibody sequences for which the HIC retention time (RT) was measured to learn the HW weights using logistic regression. We re-implemented their method but replaced the random forest regressor with a deep convolutional neural network NanoNet,^23^ a structure prediction method for nanobodies, and included more structures taken from the SAbDab database.^24^ Since this architecture is able to accurately predict the structure of nanobodies, we hypothesized it could predict the per-residue SASA of the VH of antibodies. We retrained the model from scratch while modifying the last layer of the network to output a single value corresponding to the SASA score for each residue instead of the 15 values corresponding to the coordinates of the backbone residues (see Methods A). We also trained a second version of the model that takes as input both the VH and VL of the antibodies to predict their per-residue SASA. Using the output of this model, we built a secondary solubility score that considers both VH and VL by taking the sum of the solubility scores (Eq 4) of both chains.

We use a dataset containing 137 clinical-stage antibodies for which the HIC RT was computed.^1^ We used the Thera-SAbDab database^24^ to identify the antibodies in this dataset whose structures were used to train the SASA prediction model and excluded them, leaving us with 85 clinical-stage antibodies. The Spearman correlation coefficient between the HIC RT and the predicted solubility score on the 85 clinical-stage antibodies is 0.40 (Figure 2C). Using both chains to predict the solubility score leads to a degraded correlation of 0.35. The inability of that more informative model to improve performance may be an artifact of the small test set size (85 antibodies). We compare the performance of our solubility model to other models used to evaluate the solubility of antibodies (Figure 5D). Removing the SASA in the predictive models leads to a drop in the Spearman correlation coefficient to 0.15. The SASA model we use is comparable in performance to the commonly used CamSol^18^ (0.4 vs 0.33),^25^ a sequence-based model that returns a hydrophobicity score for every amino acid in the sequence that can be averaged to produce an overall score. Finally, our method performs much better on this test set than the Gravy biopython score^26^ (0.4 vs 0.05). For the generative tasks in this paper, we only design the VH of antibodies, i.e., the VL is always fixed. Therefore, since we observe no obvious improvement in the quality of the score when considering both chains, and to limit the computational cost of the solubility model, we use the model that takes into consideration only the VH.

#### Pareto optimal binders to CB-119 peptide

To generate a diverse set of antibodies binding the CB-119 peptide and optimized for solubility, we generate 20 sets of sequences with 5 different weights w=0.85,0.875,0.9,0.95,1.0 and the 4 choices of β=−1,0,1,2. We choose this set of weights for w empirically, by starting with an affinity weight of 1.0 in order to guarantee to generate sequences with a high predicted affinity to the target and then slowly increasing the solubility weight during subsequent generations until the predicted affinity of the generated sequences was significantly lower (approximately 2 orders of magnitude). We found that an affinity weight of 0.85 worked well for this purpose. Finally, we ran three additional generation procedures with affinity weights of 0.875, 0.9 and 0.95 to obtain a smooth empirical Pareto Front. The fact that the solubility weight always remains low has to do with the scales of the affinity and solubility score. These choices would most likely change if we had considered different properties to optimize. We restrict ourselves to sequences of at most six mutations away from the wildtype (WT) AB-14 (see Discussion).

We define the distance to the Pareto front as:

(5) $$d_{P}(x)=\underset{x{\;}^{\prime }\in PO}{\min}\frac{{\left({\hat{f}}_{a\mathrm{ff}}(x)-{\hat{f}}_{a\mathrm{ff}}(x{\;}^{\prime })\right)}^{2}}{\sigma_{aff}^{2}}+\frac{{\left({\hat{f}}_{s\mathrm{ol}}(x)-{\hat{f}}_{s\mathrm{ol}}(x{\;}^{\prime })\right)}^{2}}{\sigma_{sol}^{2}},$$

where $\sigma_{aff}^{2}$ and $\sigma_{sol}^{2}$ are the variances of the affinities and solubilities of the generated sequences. Top sequences are defined as the B sequences with smallest $d_{P}$, where B is the number of sequences we wish to generate and generally correspond to a budget of sequences that can be experimentally validated following the generation process. To test the reproducibility, we ran the method 3 times with different random seeds (replicates 1–3). Additionally, since our affinity model uses only 30% of the CB-119 peptide dataset, we created three different affinity models, each trained on a different 30% subset (dataset splits 1–3). This tests whether the optimal sequences generated are consistent across different training data subsets or vary based on which sequences the affinity Gaussian Process uses for inference.

**Figure 3.**
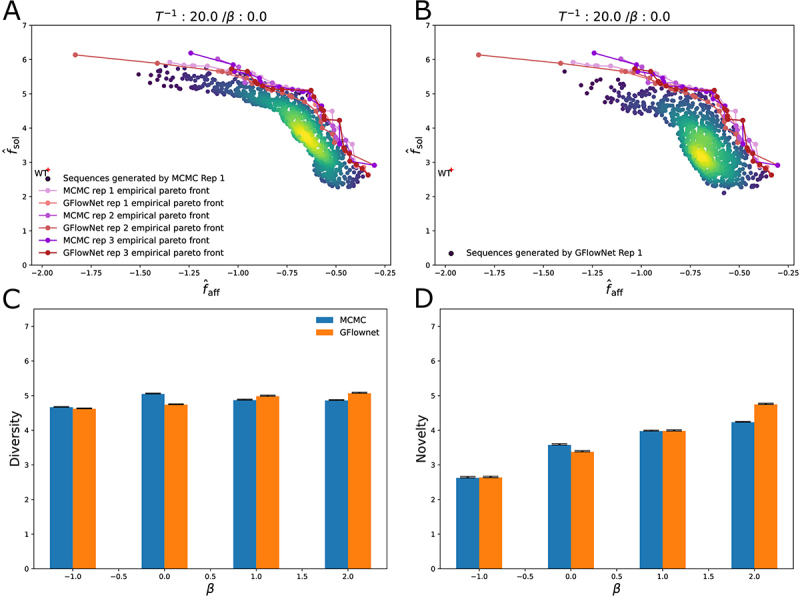
A. Density plots of 1000 sequences sub-sampled from the set generated by Metropolis-Hastings at inverse temperature $T^{-1}=20$ and β=0.0, using dataset split 1. B. Density plots of 1000 sequences sub-sampled from the set generated by the GFlowNet at inverse temperature $T^{-1}=20$ and β=0.0. C. Comparison of diversity based on generative method and choice of β. D. Comparison of novelty based on generative method and choice of β.

Figure 3 A,B show the empirical Pareto fronts from the generative process for an inverse temperature of $T^{-1}=20$, β=0, using dataset split 1 for the Gaussian Process. The Pareto fronts for β=2.0, β = 1 and β = −1 are included in SI Figure S4. The Pareto fronts generated by Metropolis-Hastings sampling and the GflowNet are similar for β=0 and β=−1, suggesting neither method is better than the other one at finding optimal sequences. For β=1 and β=2, Metropolis-Hastings sampling generates sequences with higher affinity and solubility than the GFlowNet. We hypothesized that this gap in performance by GFlowNet could be bridged by additional training. We trained a new GFlowNet model for 16,000 training steps (8000 more than previously) for β=1.0 and β=2.0 (see SI Fig S18). Our results show that extended training does not improve the networks according to the convergence measure we use (see Methods). Indeed, from 8000 to 16,000, the convergence criteria stay flat for every condition we consider. Furthermore, the empirical Pareto fronts generated by MCMC continue to dominate those of GFlowNet for both beta = 1 and beta = 2. Furthermore, we surprisingly observe that the empirical Pareto fronts of the GFlowNet trained for 16,000 steps are dominated by the empirical Pareto fronts of the GFlowNet that was trained for 8000 steps, in particular, for more soluble sequences, suggesting that more training may be harmful (perhaps due to overfitting). This suggests that the performance gap cannot be closed simply by increasing training time. Instead, more powerful neural network architectures and more thorough hyper-parameter tuning may be required to bridge the gap with MCMC. For β=0, the generated sequences that have the highest affinity have a solubility score of around 2.5 while the generated sequences that have the highest solubility have an affinity of $10^{-1.4}\approx 0.04$ nM. As a point of comparison, the 83 therapeutic antibodies on which we evaluated our solubility prediction method have an average solubility score ${\hat{f}}_{s\mathrm{ol}}$ of ≈0.7. The shape of the Pareto fronts shows a trade-off between the solubility and the affinity score. The Pareto front obtained using the predictive models suggests that increasing the solubility score from 2 to 6, a solubility score higher than any of the therapeutic antibodies used to validate the model, decreases the affinity by 1 order of magnitude.

To evaluate the novelty and diversity of a set of generated sequences $D_{gen}$, we follow Jain et al.^9^ and define a diversity index given by the mean Hamming distance within the generated dataset:

(6) $$D\mathrm{iversity}(D_{gen})=\frac{1}{N_{gen}(N_{gen}-1)}\sum_{(x,x{\;}^{\prime })\in D_{gen}}d_{H}(x,x{\;}^{\prime }),$$

where $d_{H}(x,x{\;}^{\prime })$ is the Hamming distance between x and $x{\;}^{\prime }$, and $N_{gen}=|D_{gen}|$. Novelty is defined as the mean distance to the original dataset:

(7) $$N\mathrm{ovelty}(D_{gen})=\frac{1}{N_{gen}}\sum_{x\in D_{gen}}\underset{x{\;}^{\prime }\in D_{aff}}{\min}d_{H}(x,x{\;}^{\prime }),$$

where $D_{aff}$ is the dataset of sequences for which affinity was measured. Figure 3C,D shows the diversity and novelty of the sequences generated for both methods and the three choices of β.

The average mean pairwise Hamming distance of the generated sequences is between 4.5 and 5 depending on the choice of β and sampling method (Figure 3C), compared to the maximum possible value of 12 since the exploration is limited to the space of sequences that are at most six amino acids away from the wildtype sequence. Novelty shows an expected dependence on the exploration parameter β: it ranges from ∼2.5 for a pessimistic acquisition function β=−1 to ∼4 for an exploratory acquisition function β=1. Both MCMC and GFlownet sampling methods generate a diverse and novel set of sequences. In addition, the continuity of the cloud of points shows that the methods can generate points all along the Pareto front from which we can pick the sequences with the desirable trade-off between affinity and solubility.

The inverse temperature parameter $T^{-1}$ provides a balance between diversity and optimality. A low value will lead to more diversity in the generated set but may prevent the method from generating sequences in or near the Pareto optimal set. To verify that $T^{-1}$ is sufficiently high to generate Pareto optimal sequences, we generated 30 new sets of sequences with $T^{-1}=25$ and $T^{-1}=30$ using MCMC sampling for each choice of β and w, using dataset split 1. We then combined all sequences generated for different choices of w into a single set and identified the Pareto optimal set shown in SI Fig S1. Decreasing the temperature does not generate more optimal sequences, suggesting that an inverse temperature of 20 is sufficient to generate the Pareto optimal set.

Using IgLM as a prior constrains the optimization to sequences that are more human-like. To investigate the cost of this constraint, we generated sequences without using IgLM as a prior and compared the empirical Pareto fronts to empirical Pareto fronts that use the IgLM as a prior (see SI Fig S17). We observed that Pareto fronts generated without the humanness prior slightly dominate the ones with the prior, particularly in terms of solubility, suggesting that the prior does modestly constrain the search. In exchange for this slight loss in affinity and solubility, constraining the model to generate human-like antibodies can help optimize for other important properties that can be difficult to quantify while reducing the introduction of unnatural mutations that prevent the antibody from properly folding. However, the overall trade-off appears minor, likely because all mutations in this task are restricted to the CDRs, which are more adaptable regions. Examining this trade-off in a setting where mutations occur in the framework region, where structural constraints are stronger, would be especially interesting and is a promising direction for future work.

We evaluated the sequences generated using two other developability scores reported in the previous work to evaluate generative methods^26,27^: the average charge and instability. For the instability measure, we use the instability_index() Biopython package function, which takes as input an amino acid sequence and returns a real value instability score.^28^ A protein is considered unstable if the instability score is less than −40. To estimate the charge of the antibody, we sum the contributions from all amino acids in the heavy chain sequence, adding +1 for positively charged amino acids R and K, +0.1 for all H and −1 for negatively charged amino acids D and E. A good candidate antibody should have a charge score to be between −2 and 2. The results for $T^{-1}=20.0$ and all choices of β are shown in Figure S3. The sequences generated by our method have an average charge score within the desired range, with 1.25 for β=−1 and 0.5 for β=1. The standard deviation is approximately 1 for all choices of β and we observe that a small fraction of the sequences have a charge score higher than the limit of 2. This is likely due to the fact that positively charged amino acid are more hydrophilic and the generative model can generate more soluble antibodies by adding positively charged amino acids to the antibody sequence. In addition, we observe that for all choices of β, the instability score remains on average slightly below 30 with a standard deviation of 3, with the vast majority of sequences having an instability score below the threshold of 40. In conclusion, these results suggests that our method is able to generate functional proteins, even when evaluated on methods that were not included in the multi-objective optimization function.

In addition, empirical Pareto fronts from three independent MCMC replicates are almost identical within any given train and validation split, demonstrating robustness to sampling noise. By contrast, the fronts diverge markedly across the three splits (Figure S5), although in every case the affinity-solubility trade-off persists. Accordingly, the sets of sequences generated through each split are different. Top 1000 sequences from MCMC replicate from the same split overlap by 37–58%, whereas generated sets from different splits share no more than 10% overlap (Figure S11A). Thus, the generative process is robust within, but not across, data partitions.

When using the RBF kernel, GP models will predict a high affinity to sequences that are close (in Euclidean distance), in the embedding space, to high-affinity training examples. Since those examples vary by split, so do the generated sequences. To verify this, we compared residue-frequency logos of the 15 top binders in each split and the 1 000 generated sequences per replicate, covering the 33 CDR positions (Figure S6; SI Figures S7, S8). Across all splits, the same positions tend to mutate, but specific substitutions differ, depending on what sequences were included in each split. Many solubility-enhancing hydrophilic substitutions (e.g. E or K) appear at variable sites in the training set, meaning that the generator uses those sites to balance solubility and affinity. These mutations confirm that the method does more than just copy beneficial mutations in the training set.

In summary, the generative procedure is robust to sampling variations yet sensitive to how a limited training set is partitioned. Expanding and diversifying the experimental dataset could reduce split-to-split variability and yield more consistent affinity-solubility Pareto fronts, however the amount of data required to find such a Pareto front may be prohibitive. As we will see in the next section on synthetic data, this lack of reproducibility across data splits does not prevent the method from identifying good candidate antibodies.

#### Validation on synthetic dataset

We have demonstrated the ability of energy-based generative methods to generate Pareto optimal sequences with respect to the proxy functions ${\hat{f}}_{a\mathrm{ff}}$ and ${\hat{f}}_{s\mathrm{ol}}$. However, we are not able to experimentally evaluate the $K_{d}$ of HIC of the sequences to verify that the sequences generated are actually functional. To circumvent this limitation and test the validity of the method, we designed a synthetic dataset on which to test our approach.

We define an epistatic affinity model as:

(8) $$f_{aff}(x)=f_{aff}(x^{WT})+\sum_{i=1}^{L}h_{i}(x_{i})+\sum_{i<j}J_{\mathrm{ij}}(x_{i},x_{j}),$$

where $x^{WT}$ is a wild-type sequence, $h_{i}(i)$ is the change in $\log(1/K_{d})$ after mutating the amino acid at position i to $x_{i}$ away from its wildtype value, and $h_{i}(x_{i})+h_{j}(x_{j})+J_{\mathrm{ij}}(x_{i},x_{j})$ is the change caused by mutating two amino acids at position i and j to $x_{i}$ and $x_{j}$ away from the wildtype. This epistasis model was previously used to estimate the affinity of antibodies to fluorescein based on a dataset similar in composition to the CB-119 peptide dataset studied above^29^ and fits within the broader class of epistatic models decomposed by the order of interaction, here truncated at second order-check Phillips et al.^30^ and Ranganathan review^31^ on epistasis.

Using this framework, we define two synthetic affinity models in which $h_{i}(x_{i})$ and $J_{\mathrm{ij}}(x_{i},x_{j})$ are sampled from a Gaussian distribution. The first model, referred to as the “simple” epistasis model has $H_{i}(x_{i})=N(-0.5,0.5)$ and $J_{\mathrm{ij}}(x_{i},x_{j})=N(0.0,0.5)$. The second “hard” model has $H_{i}(x_{i})=N(0.0,0.5)$ and $J_{\mathrm{ij}}(x_{i},x_{j})=N(-0.5,0.5)$. The probability that a random mutation will be deleterious is higher in the hard case than in the simple case due to the larger impact of the J terms compared to the H terms, which motivates the hard/simple terminology.

To generate our synthetic dataset, we use the same wildtype sequences as in the SARS-CoV-2 affinity dataset and compute the affinities (Eq 8) of 14,660 random variants containing all 660 single mutants, 2100 double mutants and 11,900 triple mutants, comparable to the affinity Engelhart dataset.^15^ Each mutant was generated by starting from the wildtype sequence AB-14, randomly selecting a number of positions that we want to mutate and performing a random substitution at each of these positions. We further add some random noise ∼N(0,1) to each measurement to mimic the experimental error. We generated three such datasets using different random seeds, which we refer to as dataset splits 1, 2 and 3 ($D_{syn}^{1}$,$D_{syn}^{2}$,$D_{syn}^{3}$) by analogy with the real data. We use this data to train a Gaussian Process as the new predictive affinity model ${\hat{f}}_{a\mathrm{ff}}$, and generate sequences optimized for that objective as well as ${\hat{f}}_{s\mathrm{ol}}$. For each of the two tasks, we set the inverse temperature to $T^{-1}=10$ and generate 6 sets of sequences with w∈{0.85,1.0} and β∈{−1.0,0.0,1.0,2.0}. We initially tried an inverse temperature of $T^{-1}=20$, however we found that value to be too high and the process failed to generate diverse sequences. We lowered the inverse temperature to 10 to more closely match the behavior we observed when generating binders to CB-119. For each dataset split, task and choice of β, we repeated the sampling procedure 3 times, which we refer to as replicates 1, 2 and 3.

**Figure 4.**
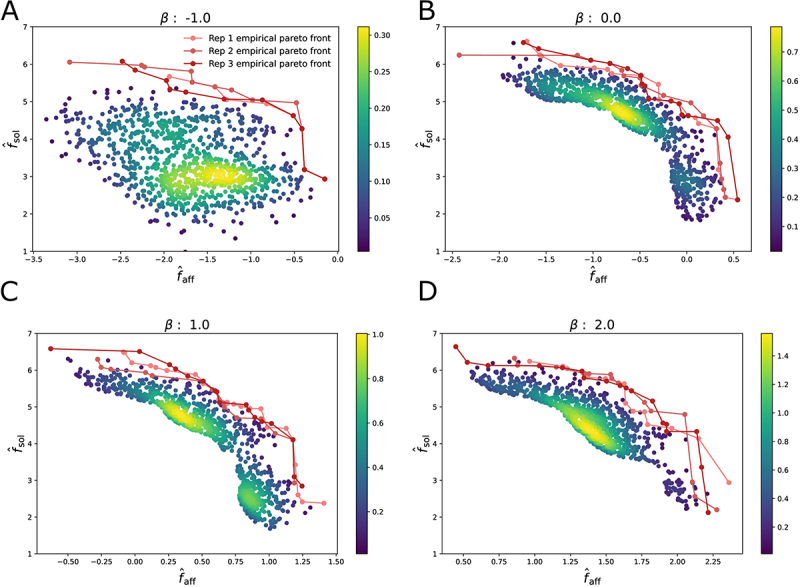
A. Density plots of 1000 sequences sub-sampled from the set generated by Metropolis-Hastings at inverse temperature $T^{-1}=10$ and β=−1.0 on the simple synthetic task using dataset split 1. B. Density plots of 1000 sequences sub-sampled from the set generated by Metropolis-Hastings at inverse temperature $T^{-1}=10$ and β=0.0 on the simple synthetic task. C. Density plots of 1000 sequences sub-sampled from the set generated by Metropolis-Hastings at inverse temperature $T^{-1}=10$ and β=1.0 on the simple synthetic task. D. Density plots of 1000 sequences sub-sampled from the set generated by Metropolis-Hastings at inverse temperature $T^{-1}=10$ and β=2.0 on the simple synthetic task.

Figure 4 shows the 1,000 sequences closest to the empirical Pareto front for the simple epistatic model for replicate 1, as well as the three empirical Pareto fronts generated for all three replicates, all for dataset split 1. These sequences were generated using MCMC sampling for the four choices of β. The results for the hard epistasis model are shown in Figure S9. Results for dataset splits 2 and 3 are also shown in Figure S9. We observe that for all choices of β, the method is able to generate a diverse set of sequences along the empirical Pareto front. As with the real data, generating sequences multiple times under the same conditions leads to finding very similar empirical Pareto fronts. We do observe that for the first dataset split, for β=−1, the method has difficulty generating diverse solutions (only 17 distinct sequences are generated); however, this is not an issue for the other dataset splits. One possible solution might be to increase the temperature for this particular setting.

We checked the overlap between the top 1000 generated sequences of each pair of dataset splits and replicated combinations (Fig. S11). Like for the real data on CB-119, we find that there is almost no overlap between sets of sequences generated using different dataset splits, although sets of sequences generated using the same dataset split have some overlap (7−16%). This suggests that this lack of overlap does not reveal issues with the generated sequences but is rather an expected feature of the procedure.

In order to investigate the usefulness of our method for antibody lead optimization, we consider the task of generating antibodies with predicted solubility score ${\hat{f}}_{s\mathrm{ol}}$ greater than a threshold $f_{sol}^{min}$ and with a synthetic affinity $f_{aff}(x)$ greater than a threshold $f_{aff}^{min}$. For each choice of β, we take the set of generated sequences using MCMC sampling and select the ones with ${\hat{f}}_{s\mathrm{ol}}>f_{sol}^{min}$. We rank the selected sequences by ${\hat{f}}_{a\mathrm{ff}}$ and keep the top B sequences, where B is the budget of sequences that can be validated with wet lab experiments. For every threshold $f_{aff}^{min}$ we count the number of sequences, out of the B sequences generated and selected, with synthetic affinity ${\hat{f}}_{a\mathrm{ff}}>f_{aff}^{min}$. We choose thresholds $f_{sol}^{min}=-\infty$ (no solubility threshold) and $f_{sol}^{min}=4.0$ to explore the differences when optimizing for both affinity and solubility vs simply affinity, and a budget of B=500. $f_{sol}^{min}=4.0$ is sufficiently high to demonstrate a difference not having a solubility threshold while still generating valid sequences. We repeated this process for each dataset split and replicate. We found that results for each replicate were very similar given each dataset split and therefore only show the results for the first replicate. The results for the simple and hard task for the first dataset split are shown in SI Figure 5. The results for the other dataset splits are included in SI Figure S12.

**Figure 5.**
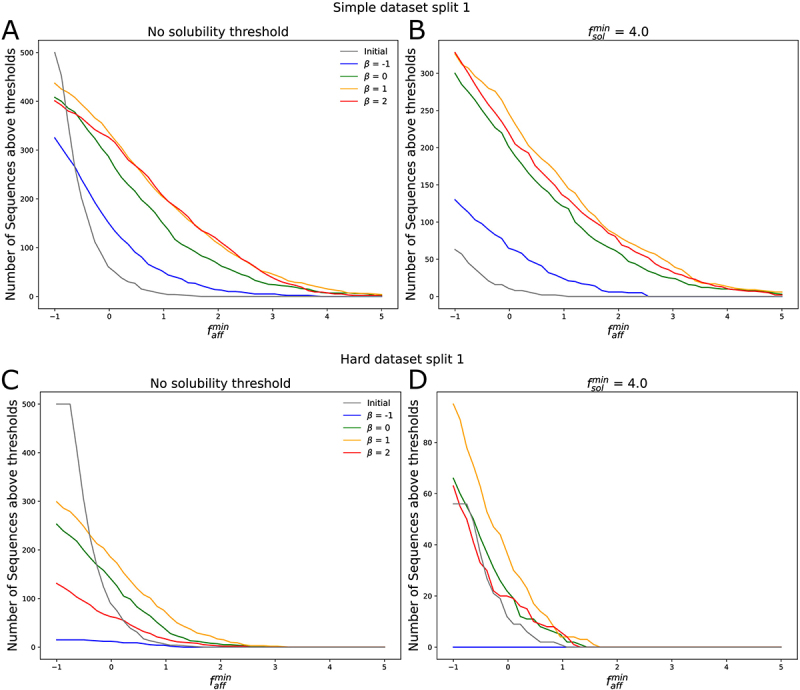
Figure A. shows the number of sequences out the top 500 selected from the set of sequences generated with Metropolis Hastings when trying to optimize the simple epistasis model with an affinity above a certain thresholds for different choices of β, using dataset split 1, for the first replicate. We compare those results to the set of sequences in the training set, indicated as initial. B shows the percentage of sequences that are above a certain threshold and also have a predicted solubility score above 4. Figure C. and D. show the results when trying to optimize the hard epistasis model.

Using the synthetic dataset, we investigate the most appropriate choice of β for lead optimization. We observe that for the simple epistatic model, both with and without a solubility threshold, the best choice was β=1. With no solubility threshold (Figure 5A,B, SI Figure S12 A,B,E,F), the best choice was β=1, although β=2 performs almost as well. While β=−1 and β=0 always perform worse, all four choices of β generate more optimal sequences than the training set. These results suggest that using an optimistic choice of β>0 helps to discover more optimal sequences, since adding additional mutations is more likely to improve the affinity.

For the hard epistatic model, where it is less likely for mutations far from the wildtype to be beneficial, the story is different. Looking at the results for the first dataset split (Figure 5C,D), we again find that using β=1.0 is the best choice, for both solubility thresholds. However, for the first split, the method fails to generate many diverse sequences for β=−1.0, which explains the low number of optimal sequences found for this choice. On the other hand, looking at dataset split 2 and 3 (SI Figure S12 C,D,G,H), we find that for both solubility thresholds it is best to use a pessimistic β=−1. However, even those choices fail to generate sequences with affinities $f_{aff}$ as high as in the simple epistatic model, dropping to 0 for $f_{aff}^{min}>1.5$. We conclude that in a setting where beneficial mutations are rare, using a pessimistic β<0 is better.

Another advantage of the energy-based framework is the possibility to balance optimality with diversity of the sequences generated by controlling the inverse temperature. To explore the trade-off between maximizing diversity and maintaining affinity, we generated sequences on the both synthetic task while lowering the inverse temperature $T^{-1}$ from 10 to 5 and 2. We evaluated the mean synthetic affinity of the top 500 sequences generated as well as their diversity and novelty (see SI Fig S16). The results show a clear trade-off: sequences generated with a higher inverse temperature achieve higher average synthetic affinity but exhibit lower diversity, while lowering the inverse temperature produces more diverse sequences at the cost of reduced affinity.

#### Comparison to other methods

We can use the synthetic dataset to compare the performance of our approach to previous methods against the ground truth. Khan et al.^26^ define the multi-objective optimization of antibodies as a constrained affinity optimization problem. They use a local search algorithm similar to hill climbing as the inner loop of an active learning framework to optimize the CDRH3 of antibody heavy chain in order to increase their affinity according to a synthetic affinity function computed with the software Absolut!,^32^ as well as three developability properties (net charge, repetition of amino acids and presence of a glycosylation motif). For each developability property, the algorithm requires an interval of valid values to be specified. Sequences that fall outside of these developable regions are automatically rejected by the search algorithm.

In order to compare the local search algorithm from AntBO to our approach, we re-implemented their method and ran it using two different sets of developability restrictions. In the first case, we limit the exploration to sequences with Hamming distance less than 6 to the wild type and restrict the search to sequences with $ln\;p_{HUM}\geq -120$. We chose −120 because all the sequences generated by MCMC and the GflowNet have $ln\;p_{HUM}\geq -120$. In the second case, we add the additional restriction that the sequences must have a solubility predicted score ${\hat{f}}_{s\mathrm{ol}}\geq 4$. For both cases, we ran the local search algorithm 800 times in parallel for 200 steps and kept the last sequences of each run as the set of generated sequences.

In addition, as a negative control, we also compared our method to a set of 500 randomly generated sequences $D_{rnd}$. We generated these sequences by starting with the best mutant in $D_{syn}^{1}$. We then randomly select 3 positions that have not yet been mutated. For each of these positions, we perform an amino acid substitution with an amino acid uniformly drawn from the 19 other amino acids.

**Figure 6.**
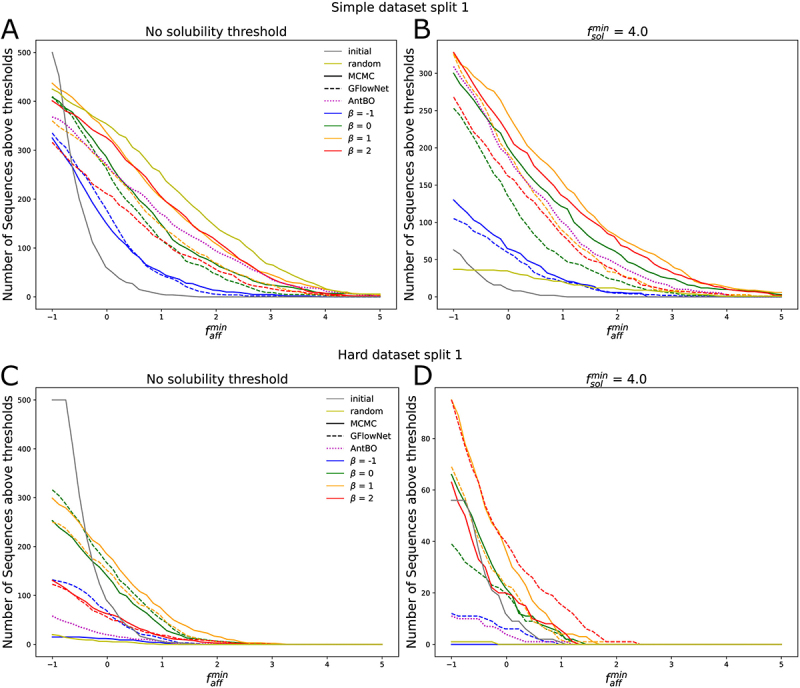
Figure A shows the number of sequences out the top 500 selected from the set of sequences generated for different choices of β and generative method (Metropolis Hastings and GFlowNet) for dataset split 1, for the first replicate. We compare those results to the set of sequences generated by the local search procedure of AntBO and the set of sequences used to train the Gaussian process affinity predictor, indicated as initial, as well as a set of randomly generated sequences in the neighborhood of the best sequence in $D_{rnd}$ indicated as random. B shows the percentage of sequences that are above a certain threshold and also have a predicted solubility score above 4. Figure C and D show the results when trying to optimize the hard epistasis model.

We compared the initial mutants $D_{syn}^{1}$, the randomly generated mutants $D_{rnd}$, and the sequences generated with the local search of AntBO to sequences generated with our method using MCMC sampling and the GflowNet (Figure 6). For both MCMC and the GflowNet, for each choice of β we combined all the sequences generated for both choices of the affinity weight w into a single set. For each set of generated sequences, we remove all sequences with $f_{\theta }^{sol}(x)<f_{sol}^{min}$ for $f_{sol}^{min}=-\infty$ and $f_{sol}^{min}=4.0$. Then, we select the top 500 sequences according to the acquisition function used during the generation process. We then compute the percentage of sequences with a synthetic affinity $f_{aff}$ higher than a chosen threshold $f_{aff}^{min}$.

For the simple epistatic model with no solubility threshold, MCMC samplings with β=1 and β=2 are the best choices of parameters and outperform the AntBO local search, except for the dataset split 2 (Figure 6A, SI Fig S13 A, E). However, we observe that the random sequences contain more optimized sequences than any of the sequences generated by our method. This suggests that the task is quite easy. For the simple epistatic model with $f_{sol}^{min}=4.0$, MCMC sampling with β=0, β=1.0 and β=2.0 outperforms the AntBO local search (Figure 6B, SI Fig S13 B, F). In addition, MCMC sampling performs better than GFlowNet for similar values of except for the second dataset split where the GFlowNet performs the best with β=2.0 than the MCMC. All of the methods do better than the random baseline and the initial dataset. For the simple epistatic model, regardless of solubility thresholds, we observe once again that using a positive β is more beneficial although if β is too high, the performance starts to decrease a little on average.

For the hard epistatic model with no solubility threshold, the best method is MCMC sampling with β=1.0 for the first dataset split and β=−1.0 for dataset split 2 and 3 (Figure 6C, SI Fig S13 C, E). As previously mentioned, the results for the first split are likely due to the small number of unique sequences generated. For the second and third split, the GflowNet with β=−1.0 performs almost as well as MCMC. Finally, both MCMC sampling and Gflownet outperform the local search of AntBO, the initial dataset and the random baseline. Finally, on the hard epistatic model with $f_{sol}^{min}=4.0$, first the first split, we find that for an affinity threshold $f_{aff}^{min}>0$, the GFlowNet with β=2.0 generates the highest number of valid sequences. For the second split (SI Fig S13 D) the GFlowNet with β=1 also performs well although both MCMC and GFlowNet with β=−1 are not far behind. Finally, for the third split, the best method is MCMC with β=−1 with the GflowNet with β=−1 also doing well. Once again, with these settings, our method outperforms AntBO, the random baseline and the initial dataset $D_{syn}^{1}$.

These results serve to demonstrate that our energy-based sampling method performs better than constrained optimization in a variety of settings. In particular, we find that the local search algorithm has more difficulty when generating sequences optimized for the hard epistasis affinity function than for the simple epistasis affinity function. In addition, we find that using optimistic choices of β is better for the simple epistatic model whereas using a pessimistic β is more appropriate when we expect that most mutations will be deleterious.

#### Validation using AlphaFold3

To further validate the method beyond synthetic data, we come back to the sequences generated from the models trained on CB-119 binding data and use AlphaFold 3 (AF3)^33^ to assess whether the designed antibodies are likely to bind their target. AF3’s ipTM score has previously been proposed as a proxy for affinity.^34^ The main idea is that AF3 can only be confident about the relative orientation of the structure of the peptide relative to the heavy and light chain if the antibody binds to the peptide.

**Figure 7.**
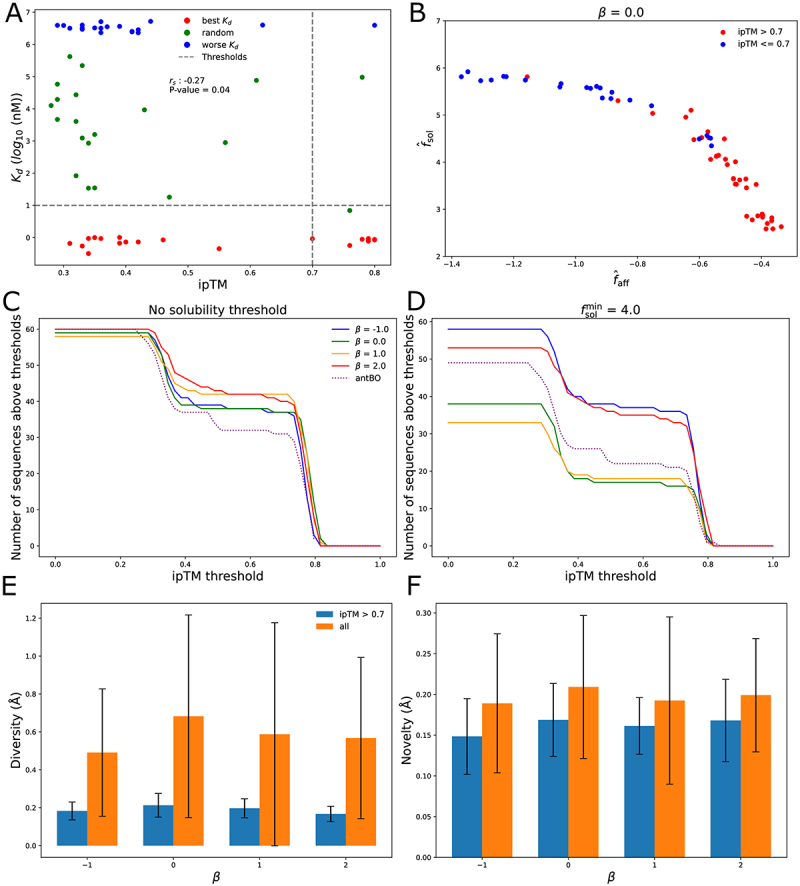
A. Scatter plots comparing the measured affinity to the CB-119 peptide ipTM of 60 antibodies selected from the first split of the CB-119 dataset. “Best” refers to the 20 sequences with the lowest $K_{d}$, “worse” the 20 sequences with the highest $K_{d}$ and “random” refers to the 20 sequences selected at random. B. Plot showing the 60 sequences closest to the empirical Pareto front generated by the first replicate for β=0. C. Plot showing the number of generated sequences with CB-119 ipTM higher than certain thresholds, using no solubility threshold. D. Plot showing the number of generated sequences with CB-119 ipTM higher than certain thresholds, using a solubility threshold of 4. E, F. Structural diversity and novelty of the 60 selected for different choices of β. We compute the diversity and novelty using Eq. (6) and (7), replacing $d_{H}$ by the RMSD of the carbon-α atoms of the CDRs of the heavy chain between structures x and x,’ after aligning them over the entire heavy chain. For the novelty, the dataset used as $D_{aff}$ contains the 60 predicted structures from the “best,” “worse” and “random” sequences.

We first tested the validity of the ipTM score as a predictor of affinity by computing it for 60 antibodies from the original CB-119 data set: the 20 top binders, the 20 worst binders, and 20 random antibody sequences. The ipTM correlated modestly with measured affinity (Spearman $r_{s}=-0.27$, Figure 7A). However, defining binders as antibodies with $K_{d}<1$ nM, and using an ipTM threshold of 0.7 for calling a positive, gives a precision of 0.81 and a recall of 0.38. We thus use the threshold of ipTM = 0.7 for calling binders to assess the performance of the method.

For each β we took the 60 sequences closest to the front (split 1, replicate 1) and scored them with AF3’s ipTM. With β = 0 (Figure 7B) the ipTM = 0.7 line cleanly separates high-affinity, lower-solubility designs ($f_{sol}<5$) from predicted non-binders but more soluble ones, implying that binders with $f_{sol}>5$ are hard to obtain. Similar trends for β = −1, 1, 2 appear in Figure SS14A-C.

Counting designs above various ipTM thresholds (with or without $f_{sol}>4$) shows that β matters little without the solubility cut (Figure 7C). With $f_{sol}>4$, β=−1 and β = 2 yield the most high-ipTM designs. Both choices appear to have an advantage over β=0 and 1 due to higher number of sequences generated with $f_{sol}>4$ (12). However, β=2.0 also generates many non-binders with high solubility scores whereas β=−1 generates very few sequences with $f_{sol}>5$. In addition, both β=−1 and β=2.0 generate more binders than the local search procedure of AntBO. Among designs with ipTM >0.7, the average pairwise CDR-loop RMSD is 0.17−0.21 Å, and the average novelty score to the predicted structures from the CB-119 dataset is 0.15–0.17 Å (Figure 7E-F).

In summary, the method generates many antibodies that are predicted to be good binders according to Alphafold 3. Despite the sequence variety, these antibodies have similar CDR conformations. To get a greater structural diversity would require a more heterogeneous training set.

## Discussion

### Data limitations and choice of models

We begin by justifying the design choices made when designing this method. First and foremost, data limitations are inherent to the task of antibody optimization. In this paper, we use a dataset that contains $10^{5}$ sequences. While this number may vary depending on the available resources, that number is usually a tiny fraction of the space of sequences we wish to explore. Even limiting the search to sequences six amino acids away from the WT, leaves $7.088\times 10^{13}$ possible candidates. Given the complexity of the task of predicting the affinity of an antibody to a target antigen based on sequence information alone, training a model on the dataset that generalizes well to the entire search space is hard. For this reason, we chose to use a Gaussian Process as this method allows us to directly estimate the epistemic uncertainty of our prediction. Furthermore, due to the limited training set size, when using a protein language model to compute the embeddings of sequences, we chose not to do any fine-tuning of the model to avoid potential over-fitting due to the large number of parameters these models use.

For the solubility model, in absence of a large exploitable dataset for machine learning, we opted to reuse the method developed by.^21^ Since their method relies on first training a SASA prediction method on publicly available structures of antibodies, we were able to implement our own SASA prediction model and then reuse the 20 hydrophobicity weights learned by^21^ on their private dataset containing 5000 pairs of antibody sequences and their HIC RT.

### Review of prior approaches

A number of methods for the generation of optimized antibody sequences have previously been proposed. Like our method, AntB0^26^ performs multi-objective optimization by modifying the heavy chain of antibodies. They seek to optimize the affinity estimate given by Absolut! as well as three developability properties. Another similar approach^35^ also performs multi-objective optimization and optimizes both affinity and thermostability. Our approach to multi-objective optimization is different from Ref.^35^ and Ref.^26^ Both of these methods define hard constraints for the developability properties. For example, AntBO requires the charge of the generated sequences to be between −2 and 2. The method in Ref.^35^ requires the predicted melting temperature to be above 60 or 65 ${\;}^{\circ }$ C. We do not use hard constraints but instead use an energy function made up of a linear combination of the predicted properties and sampled from the Boltzmann distribution defined by this energy function. The advantage of this approach is that it does not require prior knowledge of constraints for each property. In the case of the solubility prediction, for example, the HIC RT can depend on experimental conditions and defining a precise threshold for which antibodies are soluble or not is often not simple. Ref.^36^ developed an active learning loop procedure to optimize the affinity of antibodies based on the predicted free energy of the antibody-antigen complex using the Schrödinger software, using a standard multi-round active learning framework without constraining the search space. Unlike us, they do not perform multi-objective optimization, but they use a Gaussian process as a predictive model for the Schrödinger ΔΔG prediction similar to our methods and^35^ and Ref.^26^ These three methods^26,35,36^ use the expected improvement acquisition function to select which sequence to test. In contrast, we use the UCB/LCB acquisition function in order to study the effect of being optimistic vs pessimistic in a single round of optimization. Another key difference is that, we utilize a pretrained autoregressive protein language model to regularize our generative process. Of these methods, Ref.^35^ is the most similar to us in scope as it is designed to perform offline single-round multi-objective optimization, although it optimizes thermostability whereas we optimize solubility. In addition, the idea of performing the exploration in the latent space of the auto-encoder in order to amortize the exploration cost of the search space is similar to our use of the GflowNet. AntBO and Ref.^36^ on the other hand were designed for multi-round online active learning. Both methods use in-silico affinity prediction methods as $f_{aff}$, allowing them to bypass the more expensive and time-consuming wet lab experiments.

In addition,^27^ we trained a GP on the dataset of^15^ and generated new sequences using Monte Carlo Markov chain and the expected improvement acquisition function. They demonstrated that this approach is able to generate new mutants with higher affinity to the CB-119 peptide than in the initial dataset. Unlike us, however, they do not perform multi-objective optimization.

### Motivation behind investigating the choice of acquisition function

Our motivation to investigate the use of different choices of β for the acquisition function came from the fact that we have a limited dataset and the need to constrain the space of sequences on which the exploration is performed, both to facilitate the generative process and limit the search to sequences on which our predictive models are confident. By varying β, we can choose to let the generative model generate sequences farther away from the original dataset or to remain close. In previous studies,^26,35^ a trust region is used to achieve a similar goal. The trust region limits the exploration and is dynamically updated at the end of each optimization round based on whether a new best solution has been found. It is not possible to use the same approach in the context of a single round as the trust region is only modified at the end of each round. In our case, however, trying out different choices for β is a principled way of restricting the search space.

We took inspiration from recent papers in offline reinforcement learning to try both optimistic and pessimistic values of β.^37,38^ The setting of offline reinforcement learning is identical to single-round antibody optimization, wherein we do not have the ability to further query the environment or run more wet lab experiments. In this setting, multiple papers have pointed out that it may be useful to penalize uncertainty, i.e. choose a negative value for β.^39,40^

The advantage of being pessimistic is that it forces the generative model to generate sequences on which it is more confident that its prediction is accurate. This is particularly important if the number of sequences that can be tested is small and if there are few good sequences (with a high affinity) within the trust region. Instead of being optimistic and generating a large variety of sequences for which we have no guarantee that they will work, we chose to generate sequences closer to the ones in our dataset, but for which we have more accurate affinity predictions, and therefore stronger guarantees that they will bind to the target antigen.

Using the AlphaFold 3’s ipTM score for CB-119 peptide binding showed that both pessimistic and optimistic β values have benefits. Pessimistic β yielded many predicted binders among sequences with solubility scores >4.0, while optimistic β generated more highly soluble sequences, some of which were binders. This suggests that combining both approaches could be advantageous and that optimism enables simultaneous optimization of multiple properties (producing more soluble sequences), while pessimism increases the likelihood of actual binding but may make it difficult to optimize more properties.

### Limitations of our method and future research directions

There are several limitations to our study. We only consider the case of optimizing over both affinity and solubility, although there are several other important properties of interest, such as thermostability. It will be interesting to test this method when there are more properties to optimize over. Furthermore, while this approach can be extended to optimize over more than two properties, the computational cost required to generate sequences along the Pareto front and to identify the best sequences generated grows exponentially with the number of properties considered. The current approach we used of sweeping across values of w may no longer be appropriate in this situation. Investigating future solutions to this problem is an interesting area of research.

Our solubility predictive model only outputs a predictive score but no uncertainty estimates. It remains a possible research direction to build a SASA prediction model that includes uncertainty estimates. In addition, since the model outputs a prediction for every amino acid residue, it would also need to output an uncertainty estimate for every residue. It would therefore also be interesting to investigate how to combine each estimate into a single uncertainty score that could be included in the acquisition function.

We found that Pareto fronts vary with different training data and generated sequence cluster near the best training examples, suggesting that our method explores locally rather than globally despite using PLM encodings. Achieving global optimization would require larger training sets and more accurate affinity prediction models, which do not yet exist. The quality of the generated candidates depends on the training set quality – better input sequences yield better outputs according to predictive models. While different dataset splits produce different sequences, our method consistently outperforms others in generating candidates with desirable properties. However, AlphaFold 3 predictions show that all generated antibodies have similar structures, indicating limited structural diversity if these predictions are accurate.

Overall, our approach demonstrates the possibility to optimize several antibody properties in parallel. As real-life pharmaceutical projects aim at optimizing more than only two protein properties (affinity and solubility) before identifying a drug candidate, a direct application of this work would be to apply this approach to the optimization of five or more properties with experimental evaluations of the resulting sequences. By providing sequences fulfilling the drug developability criteria, the method described in this paper can help scientists to accelerate drug discovery projects.

## Methods

### Solubility predictor

In order to select the structures from which to build the training dataset for the solubility predictor, we took all the structures available in SabDab^24^ and clustered them by 98% similarity of the heavy chain sequence using CD-HIT.^41^ From each cluster, we selected the antibody with the longest heavy chain sequence, giving us 2648 distinct heavy chain structures. We use the Shrake-Rupley algorithm^22^ to compute the SASA of every residue in the heavy chains. We divided each computed SASA value by the maximum exposed side-chain SASA in the Ala-X-Ala peptides (where X is the amino acid for which the SASA was computed) as determined by.^42^

We use the convolutional neural network architecture used for NanoNet^23^ with an additional embedding layer added at the beginning. This layer takes as input a one-hot vector encoding of dimension L×20. The 20 values are used to determine which amino acid is present at position i. In addition, for each residue $a_{i}$, we provide the network with the distance to the first amino acid i and the distance to the last amino acid L−i.^43^ We found that providing both the forward and backward positioning information helps the network deal with sequences of different lengths. The output of the encoding layer is a matrix of dimension L×h with h=64.

The network was trained using stochastic gradient descent for 10 epochs and using a mini-batch size of 16. We use the MSE loss function:

(9) $$L_{\theta }=\frac{1}{L}\sum_{i=1}^{L}(S\mathrm{ASA}(j,x_{j};\theta )-S\mathrm{AS}A_{st\mathrm{ruct}}(j,x_{j}){)}^{2},$$

where $S\mathrm{ASA}(j,x_{j};\theta )$ is the predicted SASA of the residue at position i in the sequence x and $S\mathrm{AS}A_{st\mathrm{ruct}}(j,x_{j})$ is its true value.

### Gaussian process

The Gaussian process assumes that the output function f(x) was initially drawn at random, but in a way that similar x have similar f(x). The distribution f(x) for all x is assumed to be a multivariate Gaussian of constant mean C and covariance $k(x,x{\;}^{\prime })$, where k is called the kernel function and determines how similar the outputs of x and $x{\;}^{\prime }$ should be.

Let $X\in R^{n\times m}$ be the set of training data and $Y\in R^{n}$ associated affinity values. We assume that the data is noisy so that y(x)=f(x)+ε(x) with $\varepsilon (x)∼N(0,\sigma_{n}^{2})$. Call z is a new sequence for which we aim to make a prediction for f(z).

The joint distribution for the training data and the new sequence is also a multivariate Gaussian: p(Y,f(z)|X,z)∼N(μ,Σ) with:

(10) $$\mu =\left(\begin{array}{c} C\\ C\\ \ldots \\ C \end{array}\right),\;\;\Sigma =\left(\begin{array}{cc} k(X,X)+\sigma_{n}^{2}I_{n} & k(z,X)\\ k{\left(z,X\right)}^{T} & k(z,z) \end{array}\right).$$

k(X,X) a matrix of dimensions n×n where the (i,j) entry is $k(x_{i},x_{j})$. k(z,X) a vector of dimension n where the ith entry is equal to $k(z,x_{i})$ and $I_{n}$ is the identity matrix of dimension n.

We seek the posterior distribution:

(11) $$p(f(z)|X,Y,z)=\frac{p(Y,f(z)|X,z)}{p(Y|X)}$$

which is Gaussian with mean:

(12) $$\mu (f(z)|X,Y)=C+k(z,X{)}^{T}(K+\sigma_{n}^{2}I_{n}{)}^{-1}(Y-C),$$

and variance

(13) $$\sigma^{2}(f(z)|X,Y,z)=k(z,z)+k(z,X{)}^{T}(K+\sigma_{n}^{2}I_{n}{)}^{-1}k(z,X).$$

The posterior distribution for the noisy prediction y(z)=f(z)+ε(z) has an additional $\sigma_{n}^{2}$ term in its variance.

The model provides both an estimate for the affinity of a new sequence as well as an uncertainty estimate. This uncertainty σ only depends on the training data X and can be reduced by adding more training examples. Therefore, it captures the “epistemic” uncertainty of the error. On the other hand $\sigma_{n}$ is independent of the training set and z, therefore it captures the “aleatoric” uncertainty.

We use the common radial basis function (RBF) Kernel:

(14) $$k(x,x{\;}^{\prime })=\delta \exp(-\frac{∥x-x{\;}^{\prime }{∥}^{2}}{2\lambda^{2}}).$$

The RBF kernel^44^ encodes the bias that sequences close to one another in embedding space have similar affinities to the target antigen.

The norm $∥x-x{\;}^{\prime }∥$ is defined as the Euclidian distance in the embedding space. We tested the following embedding choices:

- One-hot vector embedding of the amino acid sequences. For a sequence of length L, this is a vector of length L×20.
- Embeddings computed by the protein language model ESM2.^45^ ESM2 is a BERT^46^ style transformer that was trained using the masked language task on general proteins. We compared the versions with 8 M, 150 M and 650 M parameters.
- Embeddings computed by Antiberty,^20^ a bert-style transformer, similar to ESM2, but trained uniquely on human antibody heavy chain sequences.

The parameters λ and δ of the Kernel $k(x,x{\;}^{\prime })$, as well as $\sigma_{n}$, are learned by minimizing the log marginal likelihood of the training data:

(15) $$\ln p(Y|X)=-\frac{1}{2}Y^{T}(K+\sigma_{n}^{2}I{)}^{-1}Y-\frac{1}{2}\ln2\pi |K+\sigma_{n}^{2}I|.$$

### Humaness model

To model humaness parameter $p_{HUM}$, we use IGLM,^11^ an auto-regressive transformer trained on 558 M human heavy chain sequences from the OAS database.^12^ Given the amino acid sequence from position 1 to i−1 denoted $x_{<i}$, the model returns a discrete probability distribution over the set of the 20 amino acids $p_{\mathrm{IGLM}}(x_{i}|x_{<i})$ and adds the next amino acid. For each sequence of amino acids, $p_{\mathrm{HUM}}$ is the probability that IGLM generates that particular sequence (obtained using the likelihood method of IGLM),

(16) $$p_{HUM}(x)=\prod_{i=1}^{n}p_{\mathrm{IGLM}}(x_{i}|x_{<i}),$$

which guarantees that only amino acid sequences that resemble human heavy chain sequences will have a high probability of being generated. Shuai et al.^11^ showed that sequences generated with IGLM have on average good developability properties when looking at solubility, aggregation and CDRH3 length.

### Energy-based model

We sample sequences using energy-based models (EBMs) by assigning an energy score E(x) to each amino acid sequence x where E(x) is low for desirable x. We would like to sample x from a probability distribution that is as close as possible to the distribution of natural antibodies, $p_{\mathrm{HUM}}$, while minimizing the mean of E(x):

(17) $$p{=}_{\pi \in \Pi }^{arg\;max}(\sum_{x}\pi (x)E(x)+TD_{KL}(\pi ||p_{HUM}),$$

where $D_{KL}$ is the Kullback-Leibler divergence. The solution of this minimization is given by the Boltzmann law, Eq. 1. In the particular case where $p_{HUM}$ is replaced by the uniform distribution, $D_{KL}$ becomes the negative entropy of π, and the problem reduces to the maximum entropy principle, where T plays the role of an inverse Lagrange parameter enforcing the mean value of E(x).

The temperature T sets the trade-off between the objectives of minimizing the quantity of interest E(x), and remaining as close to the basal distribution of antibodies. While T=0 reduces to finding the best sequences, T>0 ensures a higher diversity of antibodies that look like natural ones.

The energy function E(x) is chosen as a linear combination of multiple properties which we wish to minimize, called linear scalarization:

(18) $$E(x)=\sum_{i}w_{i}\;f_{i}(x)\;\;s.t\;\;\sum_{i}w_{i}=1.$$

Varying the weights $w_{i}$ makes it possible to explore the Pareto front in the T=0 limit, when that front is convex. For T>0, the vicinity of the front is explored while also ensuring that antibodies still look like natural ones drawn from $p_{HUM}$.

### MCMC sampling

To perform MCMC sampling of Eq. 1, we start from the WT sequence ‘GFTLNSYGISIYSDGRRTFYGDSVGRAAGTFDS,’ the concatenation of the CDRH1, CDRH2 and CDRH3 of the AB-14 sequence from the CB-119 dataset. The same wild-type sequence was also used to generate the synthetic datasets for the simple and hard synthetic affinity task. We set this sequence to be the first sequence $x_{0}$. At time k, we randomly sample a mutant sequence $x{\;}^{\prime }$ from the neighborhood of $x_{k}$. The neighborhood of $x_{k}$ is defined as the set of sequences that are at most 1 mutation away from $x_{k}$ and at most 6 mutations away from $x_{0}$. For each sequence with probability $m\mathrm{in}(1,p(x{\;}^{\prime })/p(x_{k}))$, we accept the mutation and set $x_{k+1}=x{\;}^{\prime }$, or otherwise we reject the mutation and set $x_{k+1}=x_{k}$.

We ran this process in parallel 8 times. Each time we performed MCMC sampling for a period of 20,000 time steps. To add a burn-in period, we removed all the sequences $x_{1},x_{2},\ldots x_{b}$ from the eight chains and computed the Gelman-Rubin statistic.^47^ The Gelman-Rubin statistic checks the convergence of multiple Markov Chain Monte Carlo (MCMC) chains by computing the ratio between the variance within each chain to the variance between chains, with values close to 1 indicating convergence. We then selected an initial timestep b such that the Gelman-Rubin statistic was as close to 1 as possible. If we could not find a timestep b such that the Gelman-Rubin statistic was lower than 1.1, we ran the sampling for 20,000 additional steps and repeated the process until convergence.

### GFlowNet sampling

For the autoregressive model used in our implementation of the GFlowNet, we used a ByteNet^48,49^ architecture. The network starts with an encoding layer that feeds into four ByteNet blocks. Each block uses masked convolutional layers with no dilution and a kernel size of 17. The output of these four blocks is a matrix of dimension L×H where L is the maximum sequence length and H is a hyper parameter representing the size of the encoding which we choose to be 16. We then feed to a decoder the $i^{\mathrm{th}}$ column of this matrix to which we append two values, i and n, where i is the position of the amino acid we are currently sampling and n is the Levenshtein distance between the sequence generated so far and the prefix of length i−1 of the WT sequence. We provide i to the decoder to simplify the learning process and n in order for the network to be able to learn to only generate sequences with at most 6 mutations from the wild-type sequence.

The decoder consists of a two layer multi-layer perceptron with a normalizing layer and ReLu activation layer between the first and second linear layer. The output of the second layer is a vector of dimension 20 containing the logits for each of the 20 amino acids.

For each GflowNet trained, we use a starting learning rate for the parameters of the network of $10^{-3}$, except for the learning rate of $Z_{\theta }$ which is set to $10^{-2}$ (an order of magnitude higher per the recommendation of the GflowNet paper). We train each network for 8000 training steps, halve the learning rate after 4000 steps and use a replay buffer with a max size of 20,000. We initialize to contain the sequences in the COVID dataset for the COVID task and the synthetic dataset for the simple and hard synthetic tasks. At each step, we first generate 16 sequences using the generative model and compute their energy E. We then sample 16 more sequences and their score from the replay buffer and update the parameters of the model using stochastic gradient descent on the set of 32 sequences. The 16 generated sequences are then added to the replay buffer and the process is repeated.

Unlike for the MCMC, there is no principled way to check that the training of the neural network has converged to a local optimum. In order to verify that the network is learning to properly generate sequences with a probability proportional to their reward, we take inspiration from Ref.^50^ Using a separate dataset of sequences from the sequences used to initiate the replay buffer, we periodically, during the training process, compute the log probability of the sequences being generated. We compute the Spearman R correlation score between the scores and the log probability of the generated sequences.

While this method has its flaws, we found that it was a simple method to verify that the generative model was correctly learning for each choice of β, w and inverse temperature $T^{-1}$ (SI Figure S2).

## Alphafold 3 predictions

Here, we provide step-by-step instructions to reproduce our Alphafold three predictions. Datasets containing the sequences of the antibody and the antigen should be downloaded at: https://github.com/statbiophys/ABGen/tree/master/lib/dataset/af3_batch_jobs_generated_beta:-1.0.json,https://github.com/statbiophys/ABGen/tree/master/lib/dataset/af3_batch_jobs_generated_beta:0.0.json,https://github.com/statbiophys/ABGen/tree/master/lib/dataset/af3_batch_jobs_generated_beta:1.0.json,https://github.com/statbiophys/ABGen/tree/master/lib/dataset/af3_batch_jobs_generated_beta:2.0.json,https://github.com/statbiophys/ABGen/tree/master/lib/dataset/af3_batch_jobs_generated_antBO_beta:0.0.json (one file for each value of β, in addition to the antBO prediction).

After logging into the server (a Google account is required), click on ‘upload json’ and select one of the downloaded json files on your local computer. After loading the json files, each job must be submitted manually, with a limit of 30 jobs per day.

## Supplementary Material

figureS2.jpg

figureS8.jpg

figureS5.jpg

figureS13.jpg

figureS6.jpg

figureS1.jpg

figureS15.jpg

figureS4.jpg

figureS9.jpg

figureS17.jpg

figureS12.jpg

figureS14.jpg

figureS11.jpg

figureS10.jpg

figureS16.jpg

figureS7.jpg

figureS3.jpg

figureS18.jpg

## Data Availability

The code and data supporting the findings of this study are available at the following GitHub repository https://github.com/statbiophys/ABGen and 10.5281/zenodo.15571259. The results generated with AlphaFold 3 were produced after the main author left Sanofi employment and Sanofi never had access to AlphaFold 3 outputs.

## References

[1] Jain T, Sun T, Durand S, Hall A, Houston NR, Jh N, Sharkey B, Bobrowicz B, Caffry I, Yu Y. Biophysical properties of the clinical-stage antibody landscape. In: Wells J, editor. Proceedings of the National Academy of Sciences; Vol. 114. 2017. p. 944–24. 10.1073/pnas.1616408114 PMC5293111 28096333

[2] McMahon C, Baier AS, Pascolutti R, Wegrecki M, Zheng S, Ong JX, Erlandson SC, Hilger D, Rasmussen SGF, Ring AM, et al. Yeast surface display platform for rapid discovery of conformationally selective nanobodies. Nat Struct Mol Biol. 2018;25(3):289–296. doi: 10.1038/s41594-018-0028-6. 29434346 PMC5839991

[3] Ledsgaard L, Kilstrup M, Karatt-Vellatt A, McCafferty J, Laustsen AH. Basics of antibody phage display technology. Toxins. 2018;10(6):236. doi: 10.3390/toxins10060236. 29890762 PMC6024766

[4] Ye W, Liu X, He R, Gou L, Lu M, Yang G, Wen J, Wang X, Liu F, Ma S, et al. Improving antibody affinity through in vitro mutagenesis in complementarity determining regions. J Biomed Res. 2022;36(3):155. doi: 10.7555/JBR.36.20220003. 35545451 PMC9179109

[5] Hearty S, Leonard P, O’Kennedy R. In: Nevoltris D, Chames P, editors. Antibody Engineering: Methods Protocols, 2nd ed. New York, NY: Humana Press; 2012. p. 411–442.

[6] Khetan R, Curtis R, Deane CM, Hadsund JT, Kar U, Krawczyk K, Kuroda D, Robinson SA, Sormanni P, Tsumoto K, et al. Current advances in biopharmaceutical informatics: guidelines, impact and challenges in the computational developability assessment of antibody therapeutics. Vol. 14. Taylor & Francis; 2022. 10.1080/19420862.2021.2020082 PMC8812776 35104168

[7] Mason DM, Friedensohn S, Weber CR, Jordi C, Wagner B, Meng SM, Ehling RA, Bonati L, Dahinden J, Gainza P, et al. Optimization of therapeutic antibodies by predicting antigen specificity from antibody sequence via deep learning. Nat Biomed Eng. 2021;5(6):600–612. doi: 10.1038/s41551-021-00699-9. 33859386

[8] Biswas S, Khimulya G, Alley EC, Esvelt KM, Church GM. Low-n protein engineering with data-efficient deep learning. Nat Methods. 2021;18(4):389–396. doi: 10.1038/s41592-021-01100-y. 33828272

[9] Jain M, Bengio E, Hernandez-Garcia A, Rector-Brooks J, Dossou BF, Ekbote CA, Fu J, Zhang T, Kilgour M, Zhang D, et al. Biological sequence design with GFlowNets. 2022: 9786–9801.

[10] Bennett NR, Watson JL, Ragotte RJ, Borst AJ, See DL, Weidle C, Biswas R, Shrock EL, Leung PJ, Huang B, et al. Atomically accurate de novo design of antibodies with RFdiffusion. Nature. 2025. 10.1038/s41586-025-09721-5 PMC12727541 41193805

[11] Shuai RW, Ruffolo JA, Gray JJ. IgLM: Infilling language modeling for antibody sequence design. Cell Systems. 2023;14:979–989.e4. 37909045 10.1016/j.cels.2023.10.001 PMC11018345

[12] Olsen TH, Boyles F, Deane CM. Observed antibody space: a diverse database of cleaned, annotated, and translated unpaired and paired antibody sequences. Protein Sci. 2022;31(1):141–146. doi: 10.1002/pro.4205. 34655133 PMC8740823

[13] Haarnoja T, Tang H, Abbeel P, Levine S. Reinforcement learning with deep energy-based policies. Proceedings of the 34th International Conference on Machine Learning; Sydney, Australia. 2017;70:1352–1361.

[14] Bengio Y, Lahlou S, Deleu T, Hu EJ, Tiwari M, Bengio E. Gflownet foundations. Jour Mac Learn Rese. 2023;24.

[15] Engelhart E, Emerson R, Shing L, Lennartz C, Guion D, Kelley M, Lin C, Lopez R, Younger D, Walsh ME. A dataset comprised of binding interactions for 104,972 antibodies against a SARS-CoV-2 peptide. Sci Data. 2022;9(1):653. doi: 10.1038/s41597-022-01779-4. 36289234 PMC9606274

[16] Gardner J, Pleiss G, Weinberger KQ, Bindel D, Wilson AG. Gpytorch: blackbox matrix-matrix Gaussian process inference with GPU acceleration. Adv Neural Inf Process Syst. 2018;327587–7597.

[17] Auer P, Cesa-Bianchi N, Fischer P. Finite-time analysis of the multiarmed bandit problem. Mach Learn. 2002;47(2–3):235–256. doi: 10.1023/A:1013689704352.

[18] Sormanni P, Aprile FA, Vendruscolo M. The CAMSOL method of rational design of protein mutants with enhanced solubility. J Mol Biol. 2015;427(2):478–490. doi: 10.1016/j.jmb.2014.09.026. 25451785

[19] Lin Z, Akin H, Rao R, Hie B, Zhu Z, Lu W, Smetanin N, Verkuil R, Kabeli O, Shmueli Y, et al. Evolutionary-scale prediction of atomic-level protein structure with a language model. Science. 2023;379(6637):1123–1130. doi: 10.1126/science.ade2574. 36927031

[20] Ruffolo JA, Gray JJ, Sulam J. Deciphering antibody affinity maturation with language models and weakly supervised learning. Patterns. 2022;3:7.

[21] Jain T, Boland T, Lilov A, Burnina I, Brown M, Xu Y, Vásquez M. Prediction of delayed retention of antibodies in hydrophobic interaction chromatography from sequence using machine learning. Bioinformatics. 2017;33(23):3758–3766. doi: 10.1093/bioinformatics/btx519. 28961999

[22] Shrake A, Rupley JA. Environment and exposure to solvent of protein atoms. lysozyme and insulin. J Mol Biol. 1973;79(2):351–371. doi: 10.1016/0022-2836(73)90011-9. 4760134

[23] Cohen T, Halfon M, Schneidman-Duhovny D. Nanonet: rapid and accurate end-to-end nanobody modeling by deep learning. Front Immunol. 2022;13:958584. doi: 10.3389/fimmu.2022.958584. 36032123 PMC9411858

[24] Dunbar J, Krawczyk K, Leem J, Baker T, Fuchs A, Georges G, Shi J, Deane CM. Sabdab: the Structural Antibody Database. Nucl Acids Res. 2014;42(D1):D1140–D1146. doi: 10.1093/nar/gkt1043. 24214988 PMC3965125

[25] Sormanni P, Amery L, Ekizoglou S, Vendruscolo M, Popovic B. Rapid and accurate in silico solubility screening of a monoclonal antibody library. Sci Rep. 2017;7(1):8200. doi: 10.1038/s41598-017-07800-w. 28811609 PMC5558012

[26] Khan A, Cowen-Rivers AI, Deik DGX, Grosnit A, Robert P, Greiff V, Smorodina E, Rawat P, Akbar R, Dreczkowski K, et al. Antbo: towards real-world automated antibody design with combinatorial Bayesian optimisation. *arXiv preprint arXiv:2201.12570*.SSRN Electron J. 2022; doi: 10.2139/ssrn.4115860. PMC9939385 36814835

[27] Li L, Gupta E, Spaeth J, Shing L, Jaimes R, Engelhart E, Lopez R, Caceres RS, Bepler T, Walsh ME. Machine learning optimization of candidate antibody yields highly diverse sub-nanomolar affinity antibody libraries. Nat Commun. 2023;14(1):3454. doi: 10.1038/s41467-023-39022-2. 37308471 PMC10258481

[28] Guruprasad K, Reddy BB, Pandit MW. Correlation between stability of a protein and its dipeptide composition: a novel approach for predicting in vivo stability of a protein from its primary sequence. Protein Eng Des Sel. 1990;4(2):155–161. doi: 10.1093/protein/4.2.155. 2075190

[29] Adams RM, Kinney JB, Walczak AM, Mora T. Epistasis in a fitness landscape defined by antibody-antigen binding free energy. Cell Syst. 2019;8(1):86–93. doi: 10.1016/j.cels.2018.12.004. 30611676 PMC6487650

[30] Phillips AM, Lawrence KR, Moulana A, Dupic T, Chang J, Johnson MS, Cvijovic I, Mora T, Walczak AM, Desai MM. Binding affinity landscapes constrain the evolution of broadly neutralizing anti-influenza antibodies. eLife. 2021;10:e71393. doi: 10.7554/eLife.71393. 34491198 PMC8476123

[31] Poelwijk FJ, Krishna V, Ranganathan R. The context-dependence of mutations: a linkage of formalisms. PLOS Comput Biol. 2016;12(6):e1004771. doi: 10.1371/journal.pcbi.1004771. 27337695 PMC4919011

[32] Robert PA, Akbar R, Frank R, Pavlović M, Widrich M, Snapkov I, Slabodkin A, Chernigovskaya M, Scheffer L, Smorodina E, et al. Unconstrained generation of synthetic antibody–antigen structures to guide machine learning methodology for antibody specificity prediction. Nat Comput Sci. 2022;2(12):845–865. doi: 10.1038/s43588-022-00372-4. 38177393

[33] Abramson J, Adler J, Dunger J, Evans R, Green T, Pritzel A, Ronneberger O, Willmore L, Ballard AJ, Bambrick J, et al. Accurate structure prediction of biomolecular interactions with AlphaFold 3. Nature. 2024;630(8016):493–500. doi: 10.1038/s41586-024-07487-w. 38718835 PMC11168924

[34] Wee J, Wei GW. Evaluation of AlphaFold 3’s protein–protein complexes for predicting binding free energy changes upon mutation. J Chem Inf Model. 2024;64(16):6676–6683. doi: 10.1021/acs.jcim.4c00976. 39116039 PMC11351016

[35] Zeng Y, Elliott H, Maffettone P, Greenside P, Bastani O, Gardner JR. Antibody design with constrained Bayesian optimization. GEM workshop, International Conference on Learning Representations (ICLR). 2024.

[36] Gessner A, Ober SW, Vickery O, Oglić D, Uçar T. NeurIPS 2024 Workshop on Bayesian Decision-making and Uncertainty. 2024. *arXiv preprint arXiv:2406.07263*.

[37] Moskovitz T, Parker-Holder J, Pacchiano A, Arbel M, Jordan M. Tactical optimism and pessimism for deep reinforcement learning. Adv Neural Inf Process Syst. 2021;34:12849–12863.

[38] Xie T, Cheng CA, Jiang N, Mineiro P, Agarwal A. Bellman-consistent pessimism for offline reinforcement learning. Advances in Neural Information Processing Systems. In: Ranzato M, Beygelzimer A, Dauphin Y, Liang P Vaughan J. editors. Vol. 34. Curran Associates, Inc.; 2021. p. 6683–6694.

[39] Shi L, Li G, Wei Y, Chen Y, Chi Y. Pessimistic Q-learning for offline reinforcement learning: towards optimal sample complexity. 2022, pp 19967–20025.

[40] Koppel A, Bhatt S, Guo J, Eappen J, Wang M, Ganesh S. Information-directed pessimism for offline reinforcement learning. Proceedings of the 41st International Conference on Machine Learning. PMLR. Vol. 235. 2024.

[41] Fu L, Niu B, Zhu Z, Wu S, Li W. Cd-hit: accelerated for clustering the next-generation sequencing data. Bioinformatics. 2012;28(23):3150–3152. doi: 10.1093/bioinformatics/bts565. 23060610 PMC3516142

[42] Chennamsetty N, Voynov V, Kayser V, Helk B, Trout BL. Prediction of aggregation prone regions of therapeutic proteins. J Phys Chem B. 2010;114(19):6614–6624. doi: 10.1021/jp911706q. 20411962

[43] Sethna Z, Elhanati Y, Callan CG Jr, Walczak AM, Mora T. Olga: Fast computation of generation probabilities of B-and T-cell receptor amino acid sequences and motifs. Bioinformatics. 2019;35(17):2974–2981. doi: 10.1093/bioinformatics/btz035. 30657870 PMC6735909

[44] Williams CK, Rasmussen CE. Gaussian processes for machine learning. Vol. 2. Cambridge (MA): MIT press; 2006.

[45] Lin Z, Akin H, Rao R, Hie B, Zhu Z, Lu W, Santos Costa A, Fazel-Zarandi M, Sercu T, Candido S. Evolutionary-scale prediction of atomic-level protein structure with a language model. Science. 2023;379:1123–1130. 10.1126/science.ade2574 36927031

[46] Devlin J. Bert: Pre-training of deep bidirectional transformers for language understanding. 2018. *arXiv preprint arXiv:1810.04805*.

[47] Gelman A, Rubin DB. Inference from iterative simulation using multiple sequences. Statist Sci. 1992;7(4):457–472. doi: 10.1214/ss/1177011136.

[48] Kalchbrenner N, Espeholt L, Simonyan K, Oord AV, Graves A, Kavukcuoglu K, et al. Neural machine translation in linear time. 2016. *arXiv preprint arXiv:1610.10099*.

[49] Yang KK, Fusi N, Lu AX. Convolutions are competitive with transformers for protein sequence pretraining. Cell Syst. 2024;15(3):286–294. doi: 10.1016/j.cels.2024.01.008. 38428432

[50] Madan K, Rector-Brooks J, Korablyov M, Bengio E, Jain M, Nica AC, Bosc T, Bengio Y, Malkin N. Learning GFlowNets From Partial Episodes For Improved Convergence And Stability. Proceedings of the 40th International Conference on Machine Learning. PLMR. Vol. 202. 2023. p. 23467–23483.

