# Supplementary figures and images for: Energy-based generative models for monoclonal antibodies
Source: MAbs. 2025 Nov 25;17(1):2584935. doi: 10.1080/19420862.2025.2584935 (PMC12952271; doi:10.1080/19420862.2025.2584935)

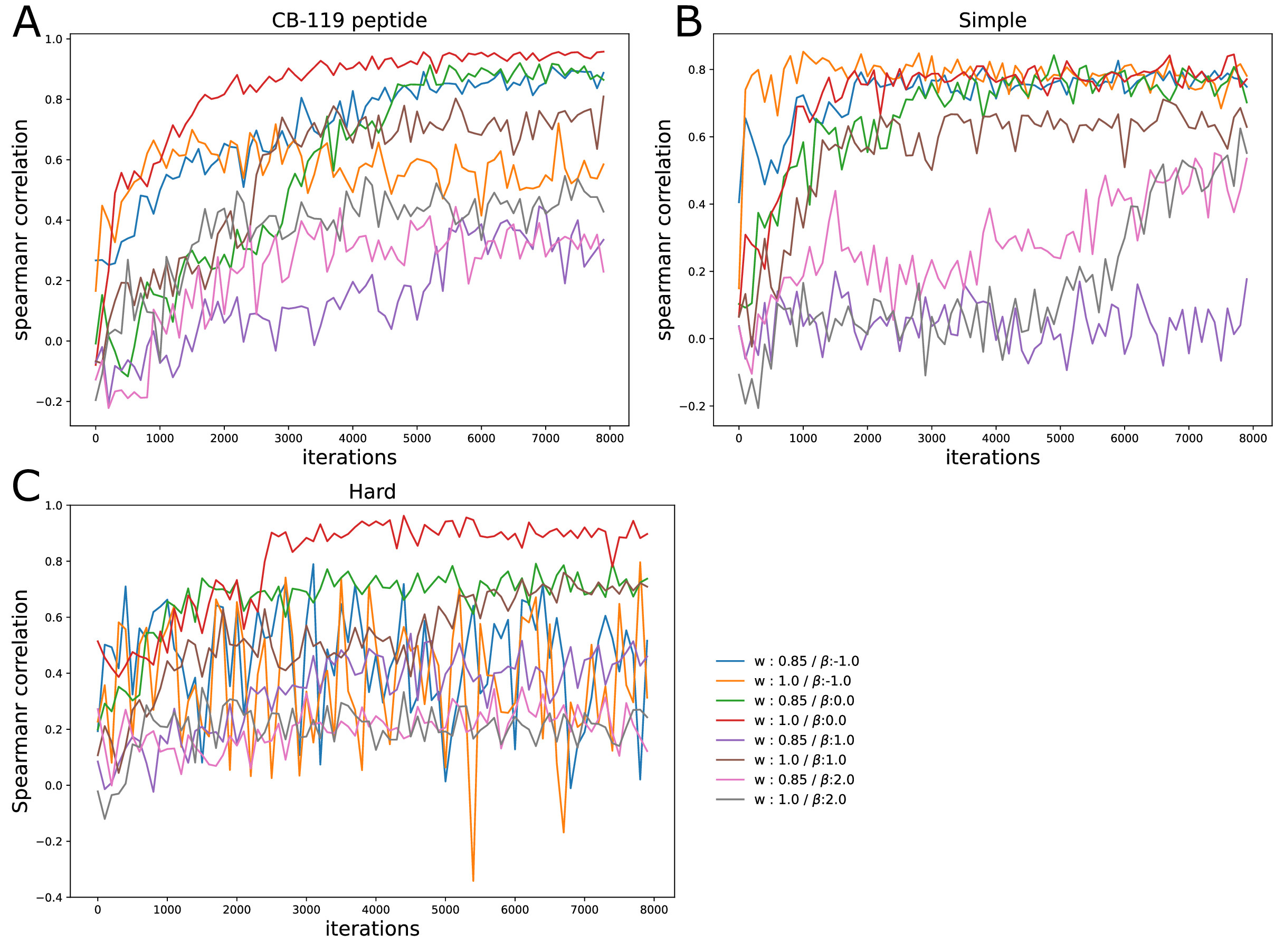

Supplement: figureS2.jpg [file KMAB_A_2584935_SM5434.jpg]

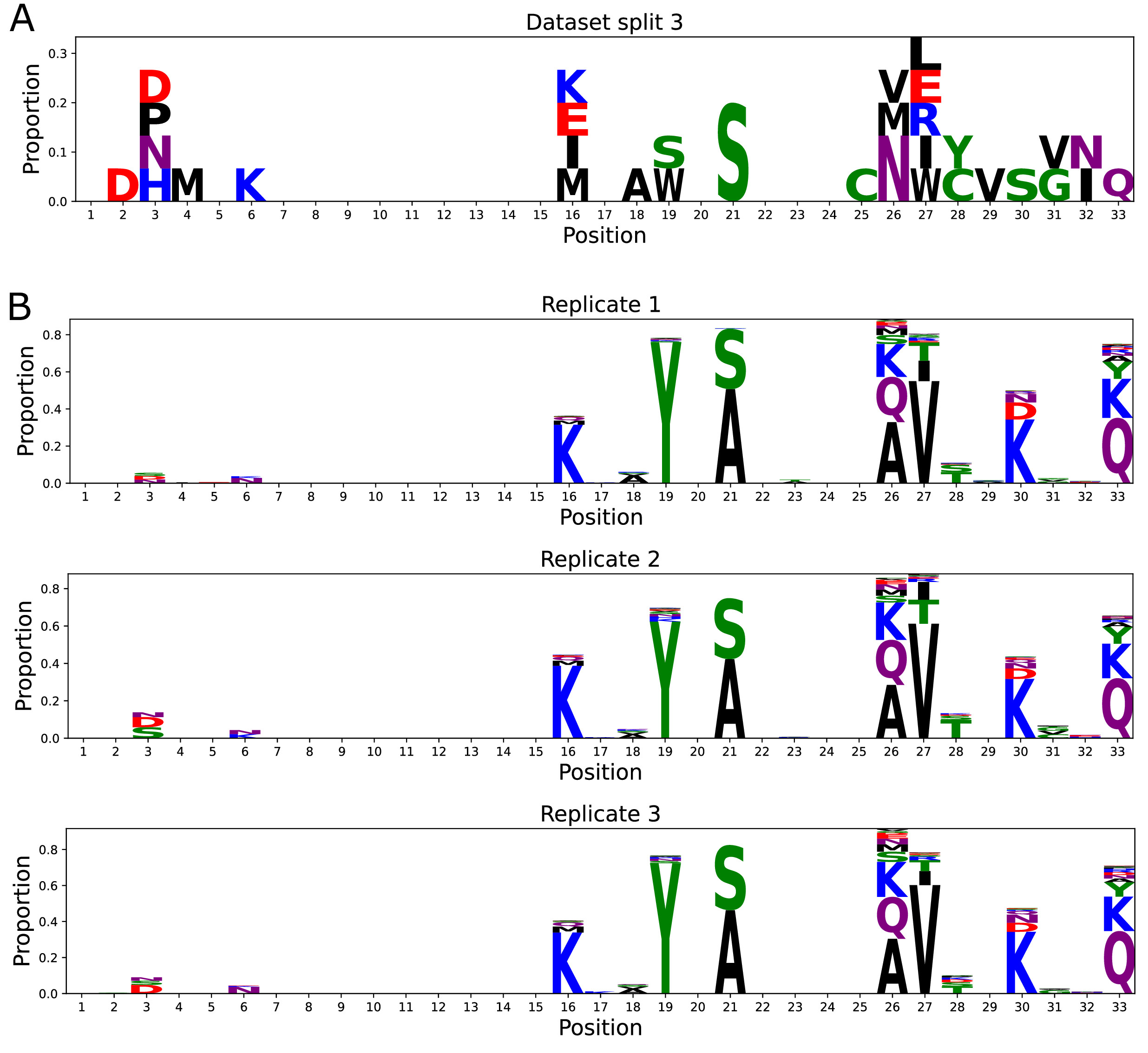

Supplement: figureS8.jpg [file KMAB_A_2584935_SM5433.jpg]

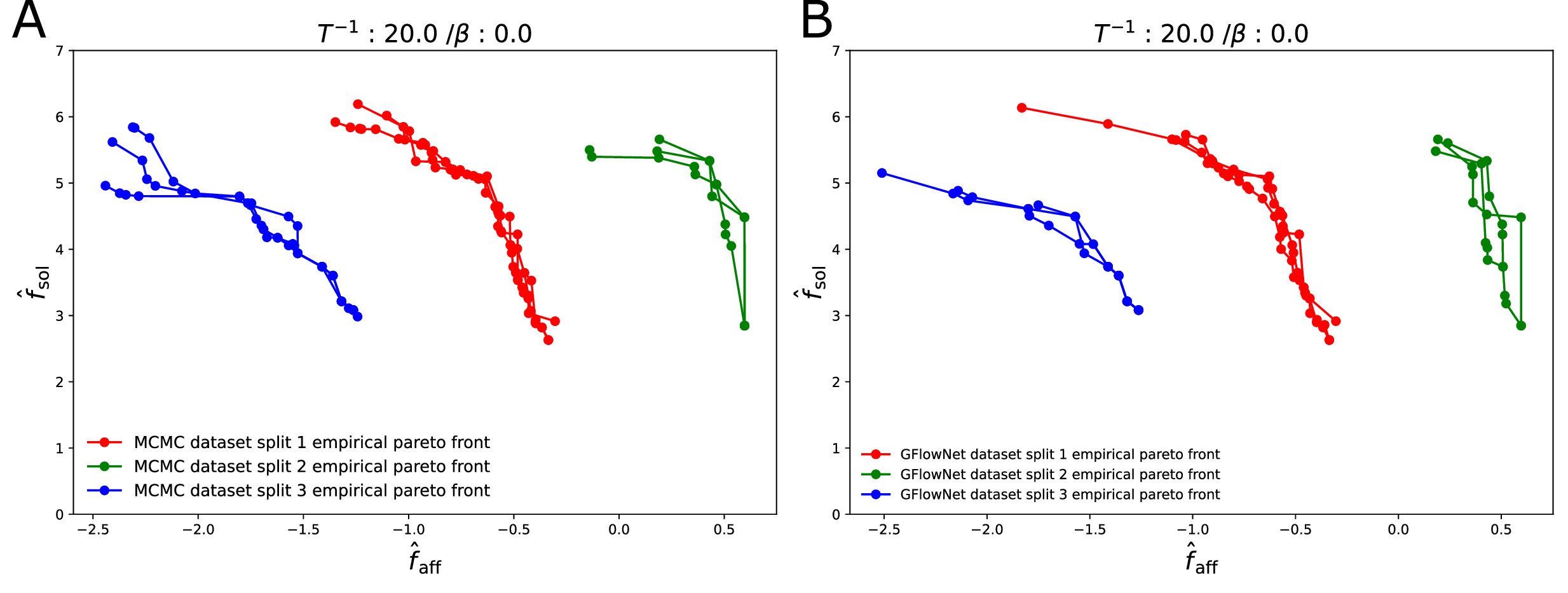

Supplement: figureS5.jpg [file KMAB_A_2584935_SM5432.jpg]

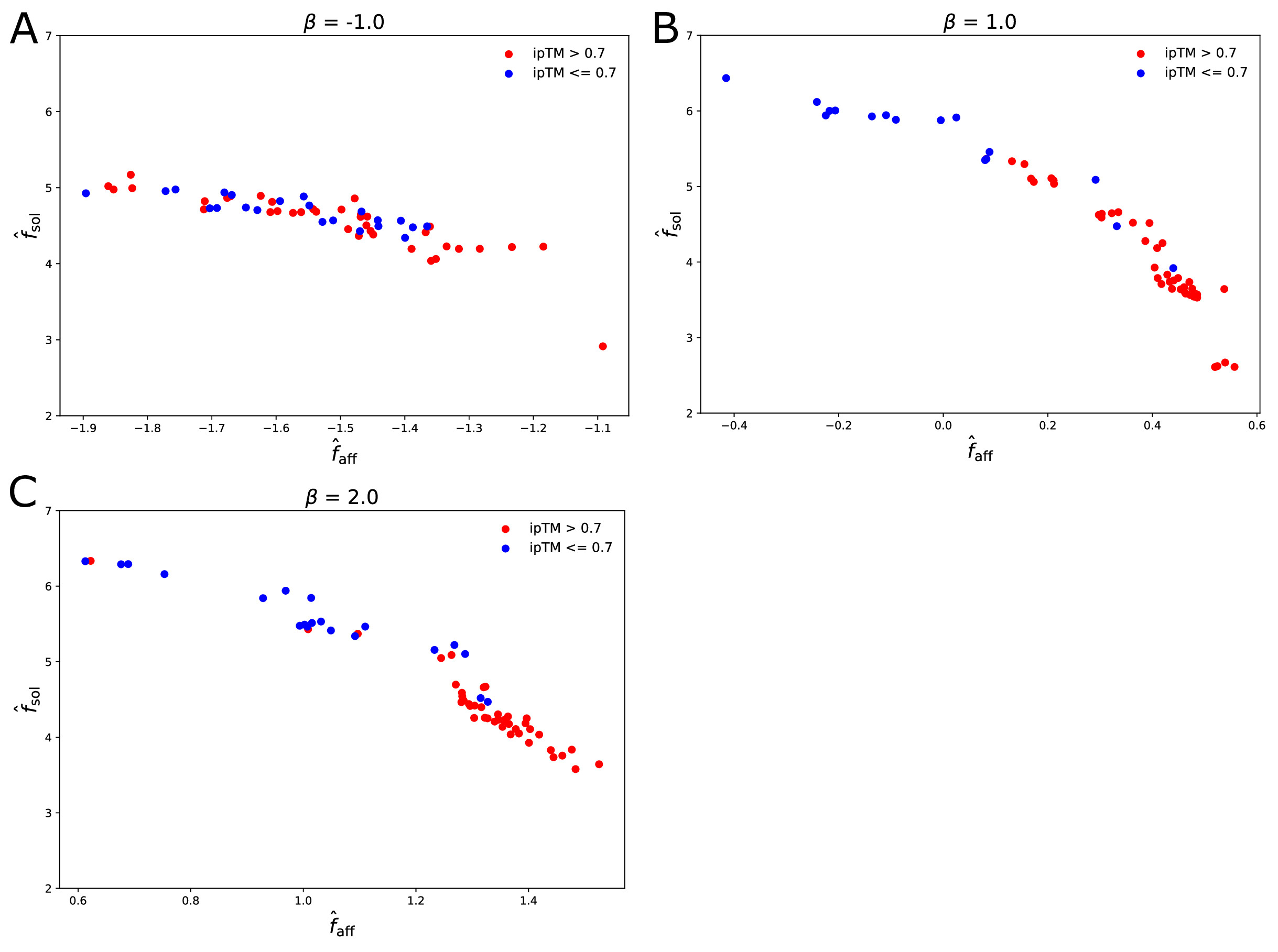

Supplement: figureS13.jpg [file KMAB_A_2584935_SM5431.jpg]

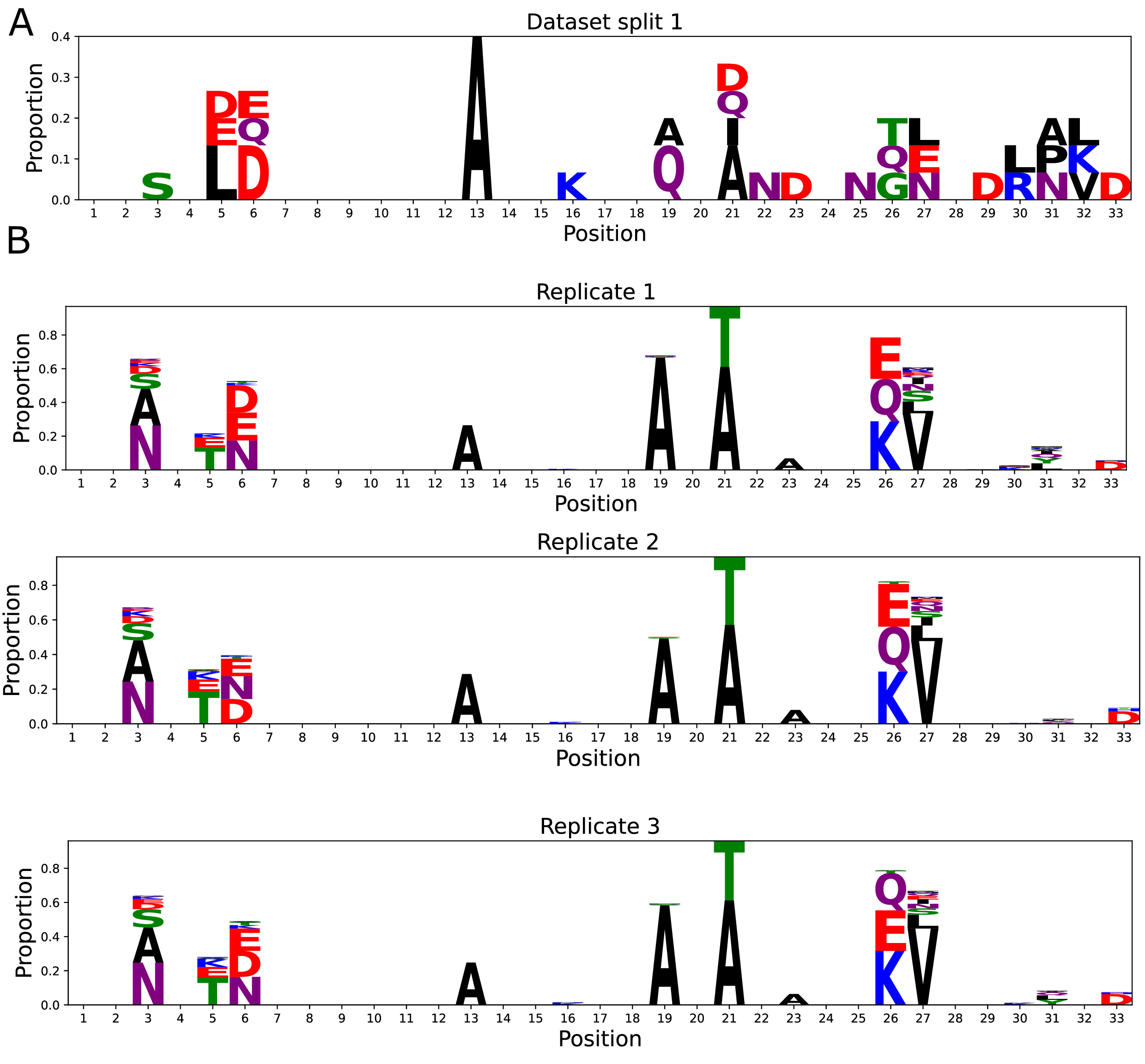

Supplement: figureS6.jpg [file KMAB_A_2584935_SM5430.jpg]

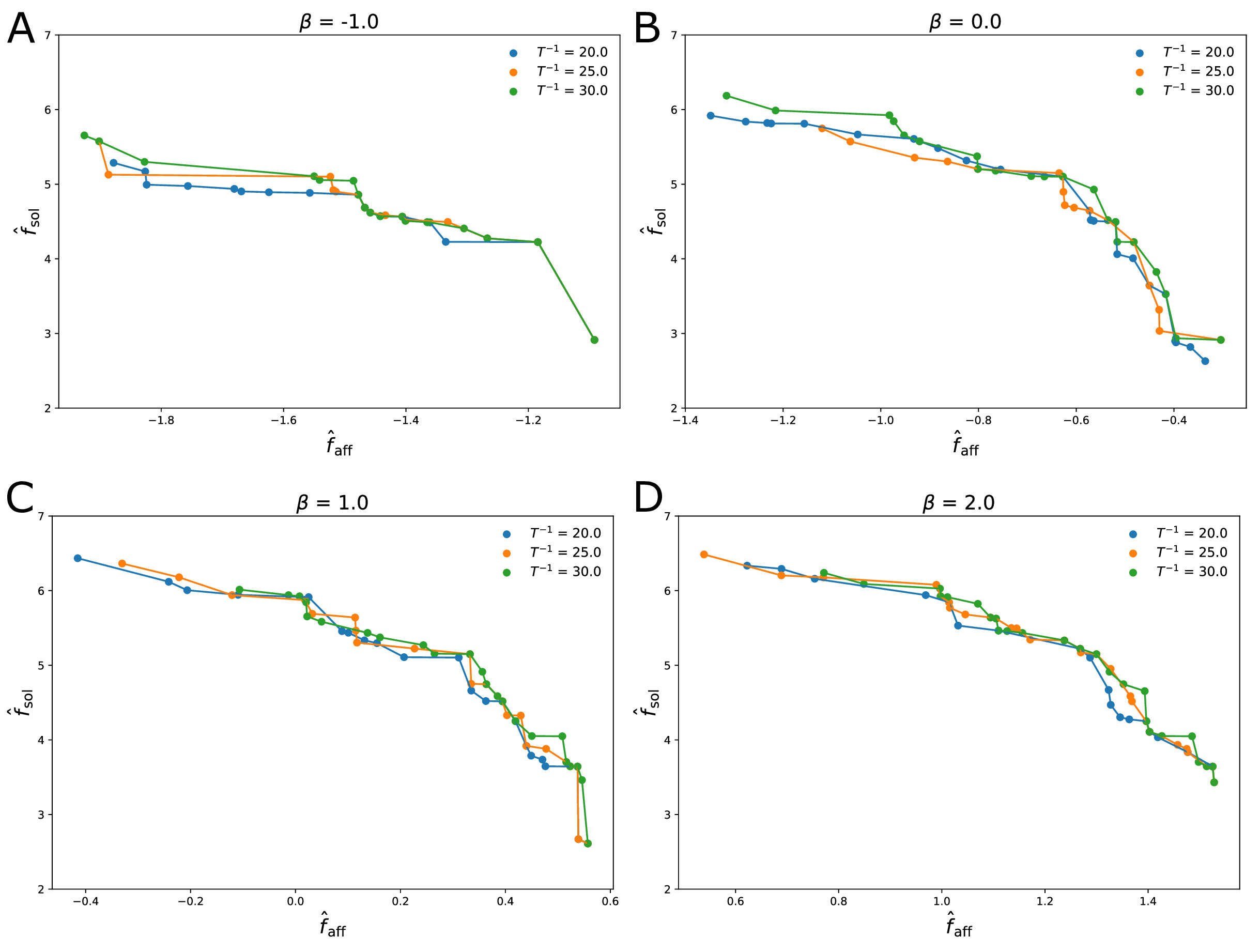

Supplement: figureS1.jpg [file KMAB_A_2584935_SM5429.jpg]

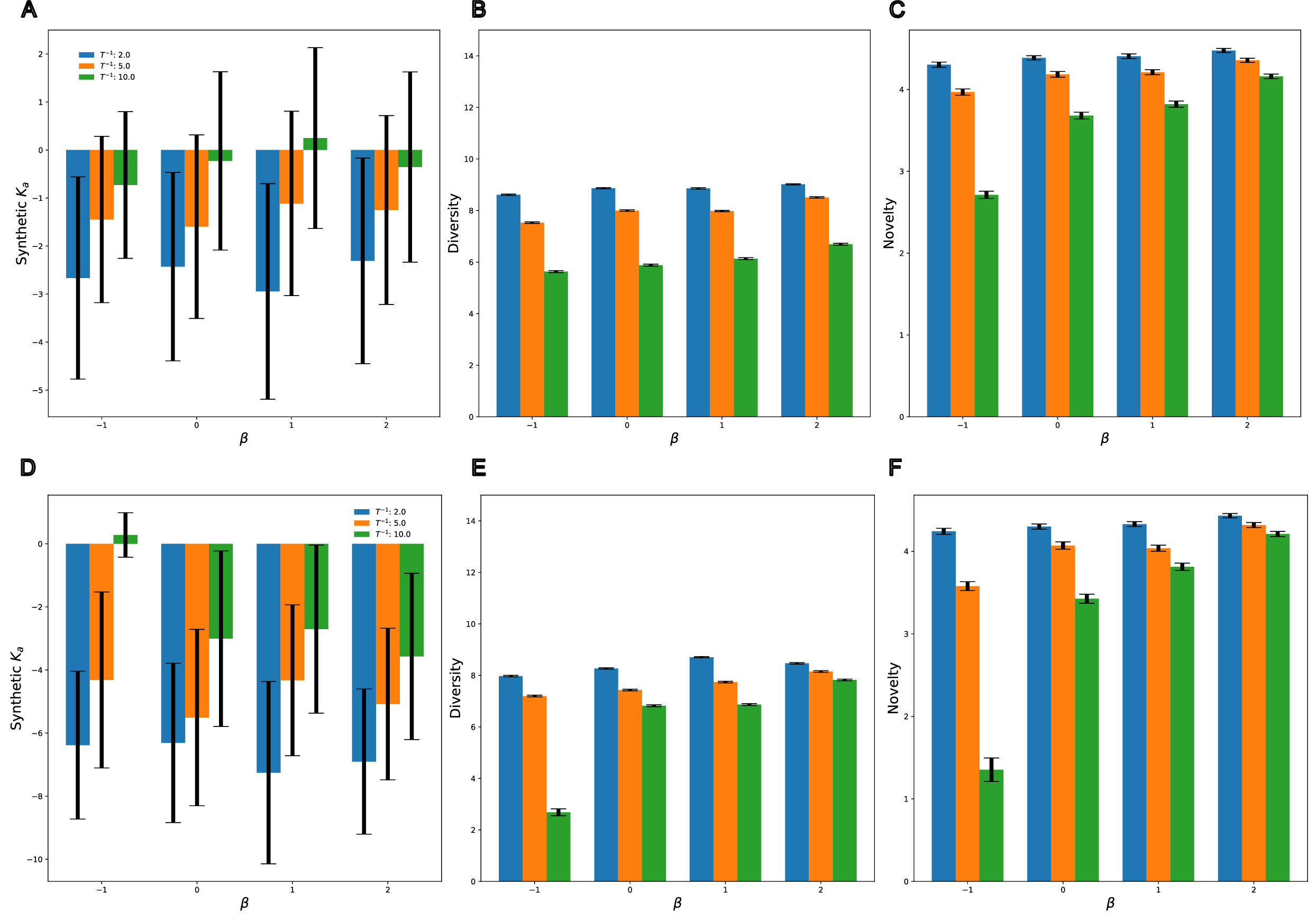

Supplement: figureS15.jpg [file KMAB_A_2584935_SM5428.jpg]

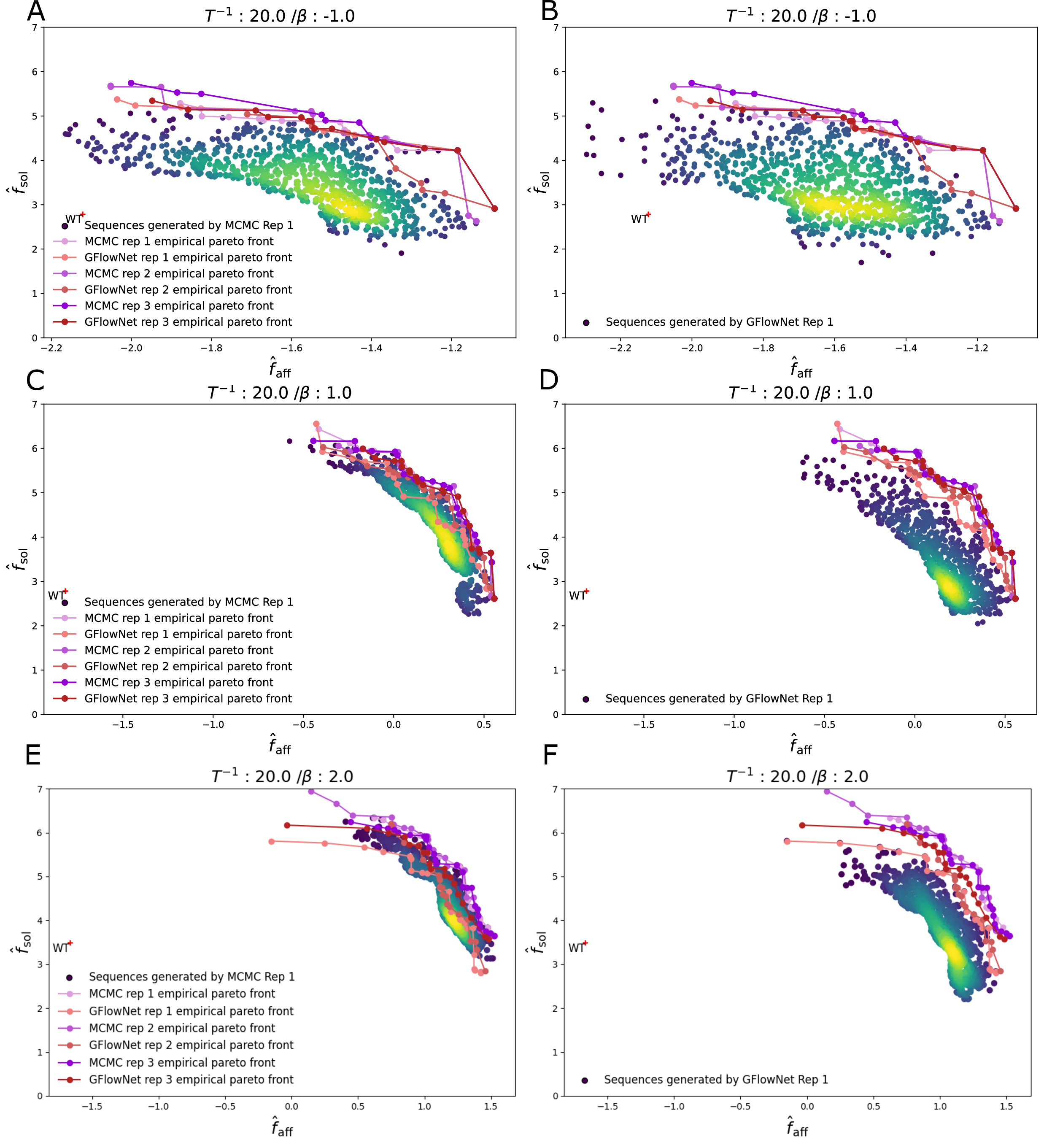

Supplement: figureS4.jpg [file KMAB_A_2584935_SM5427.jpg]

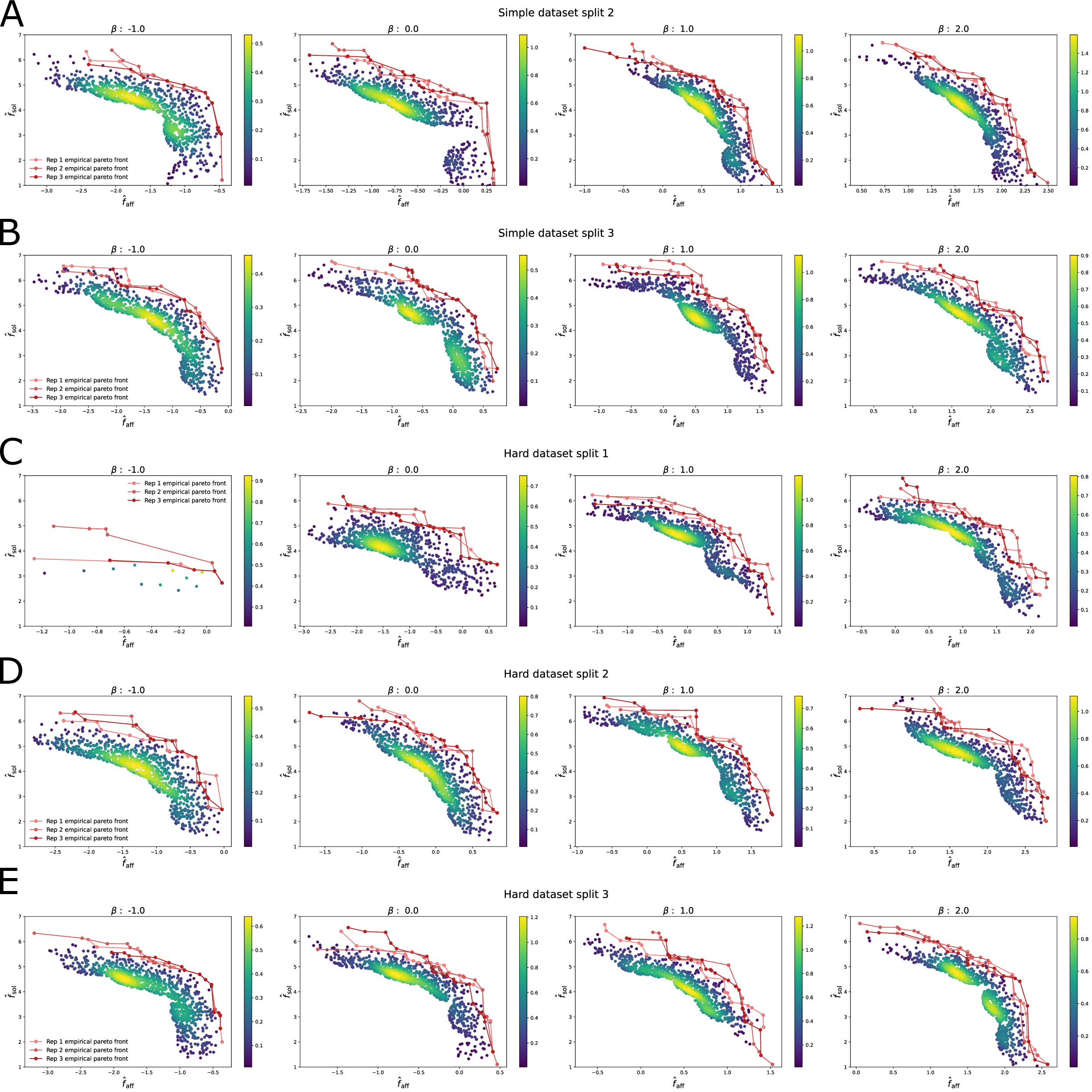

Supplement: figureS9.jpg [file KMAB_A_2584935_SM5426.jpg]

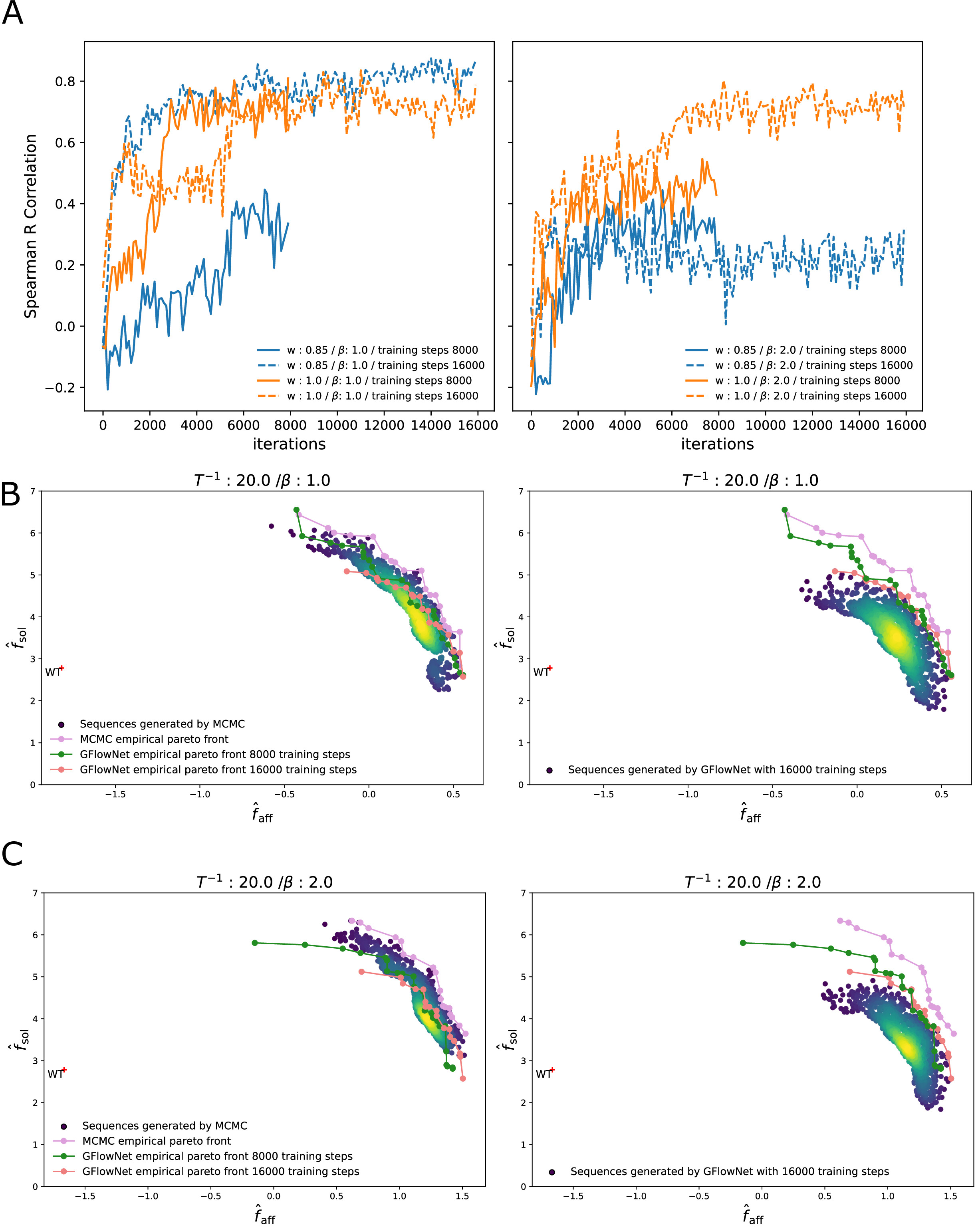

Supplement: figureS17.jpg [file KMAB_A_2584935_SM5425.jpg]

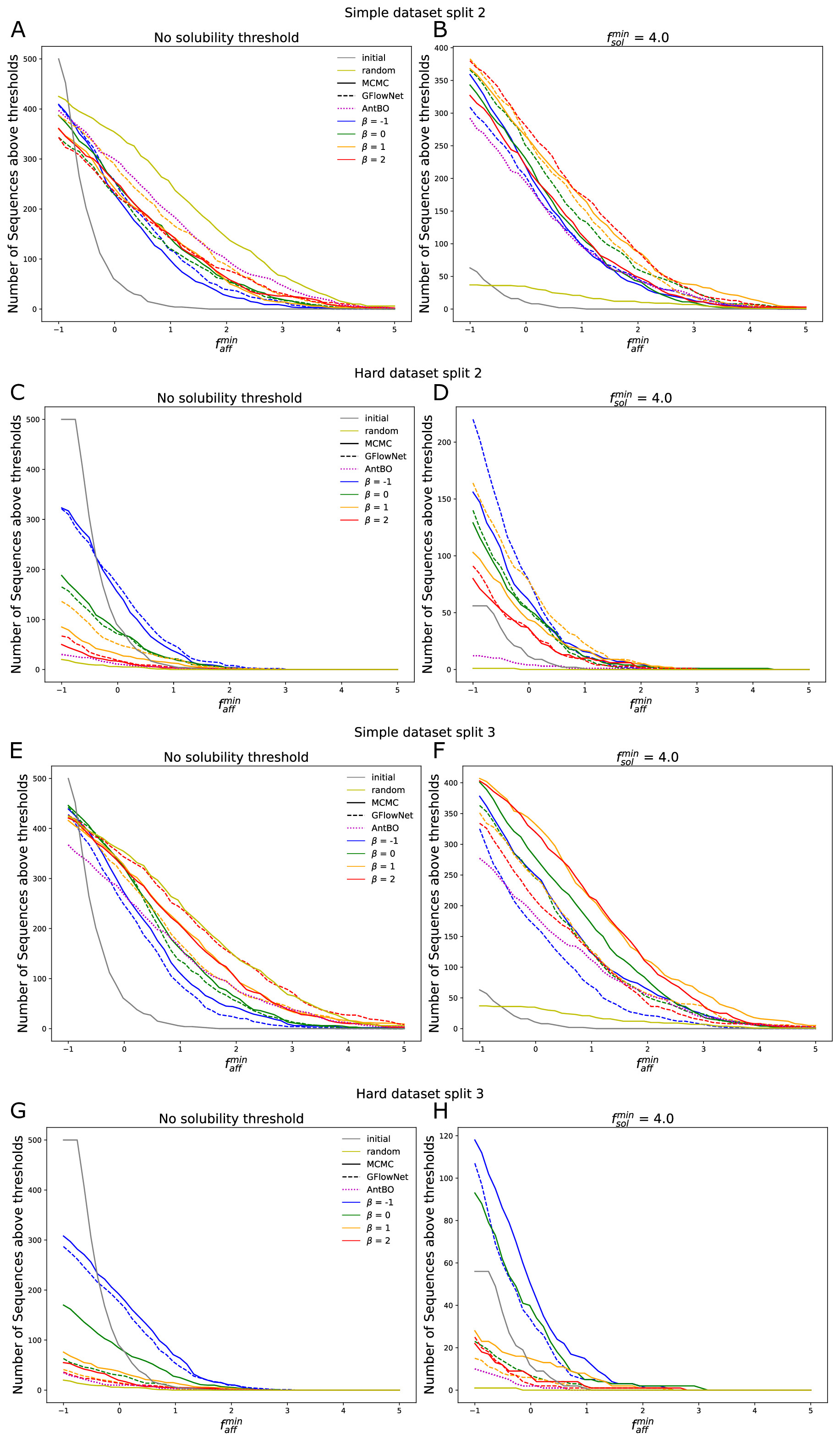

Supplement: figureS12.jpg [file KMAB_A_2584935_SM5424.jpg]

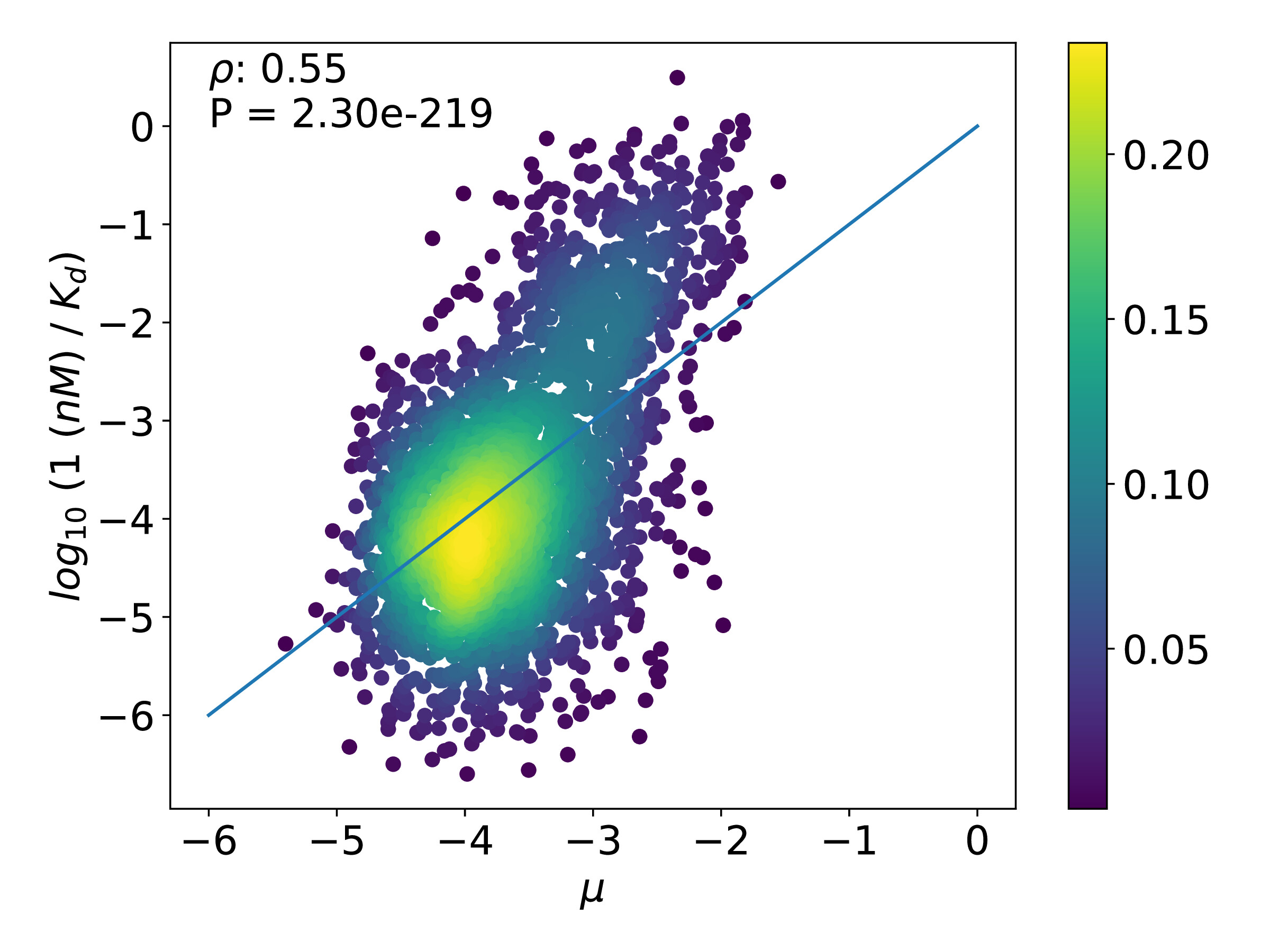

Supplement: figureS14.jpg [file KMAB_A_2584935_SM5423.jpg]

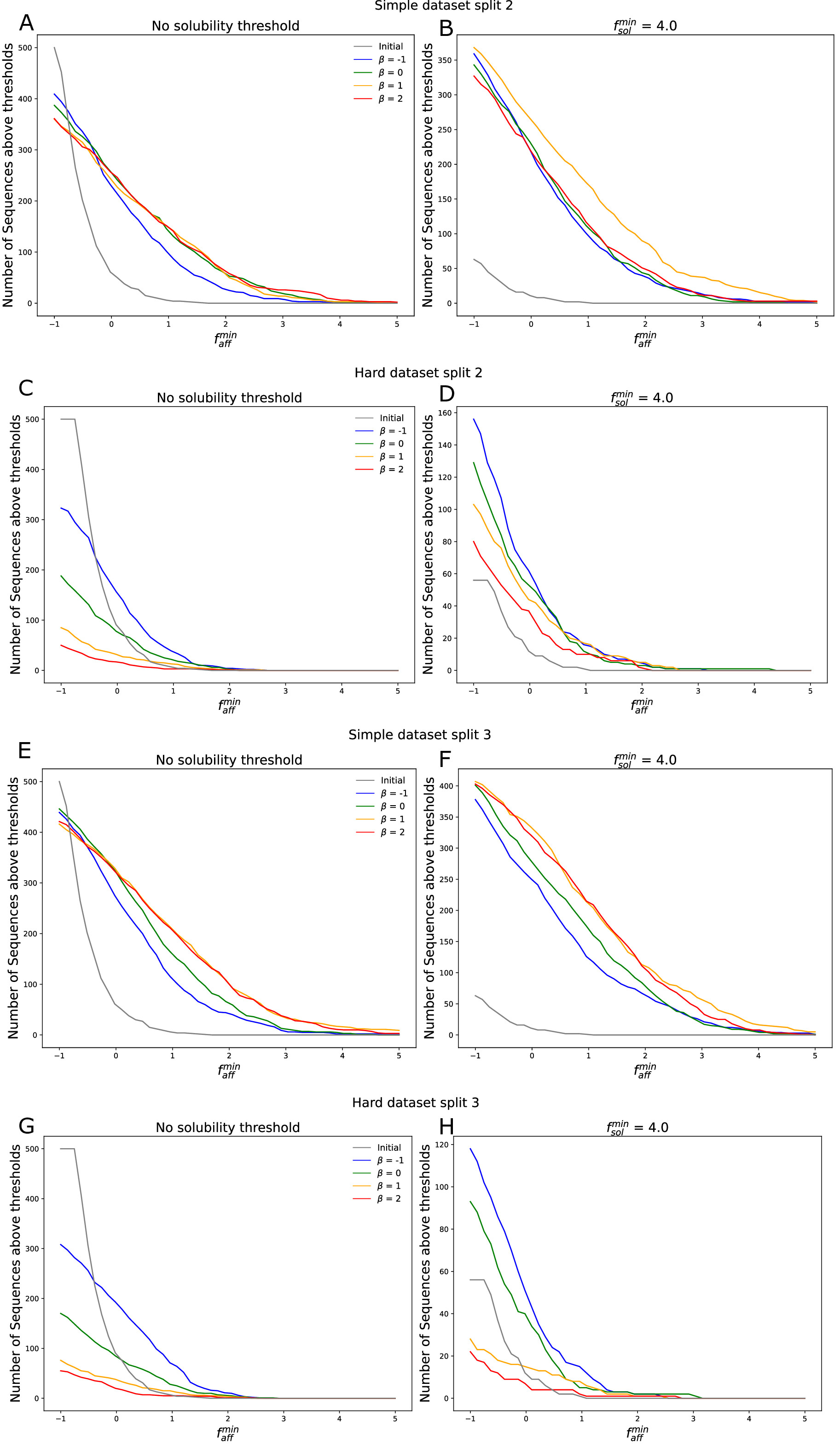

Supplement: figureS11.jpg [file KMAB_A_2584935_SM5422.jpg]

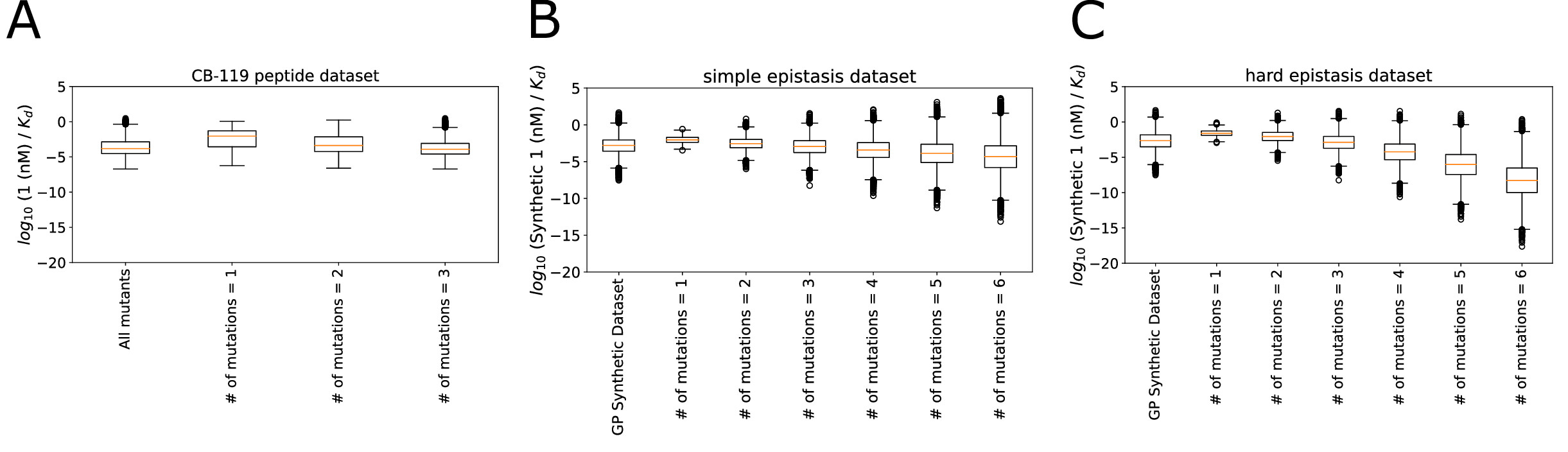

Supplement: figureS10.jpg [file KMAB_A_2584935_SM5421.jpg]

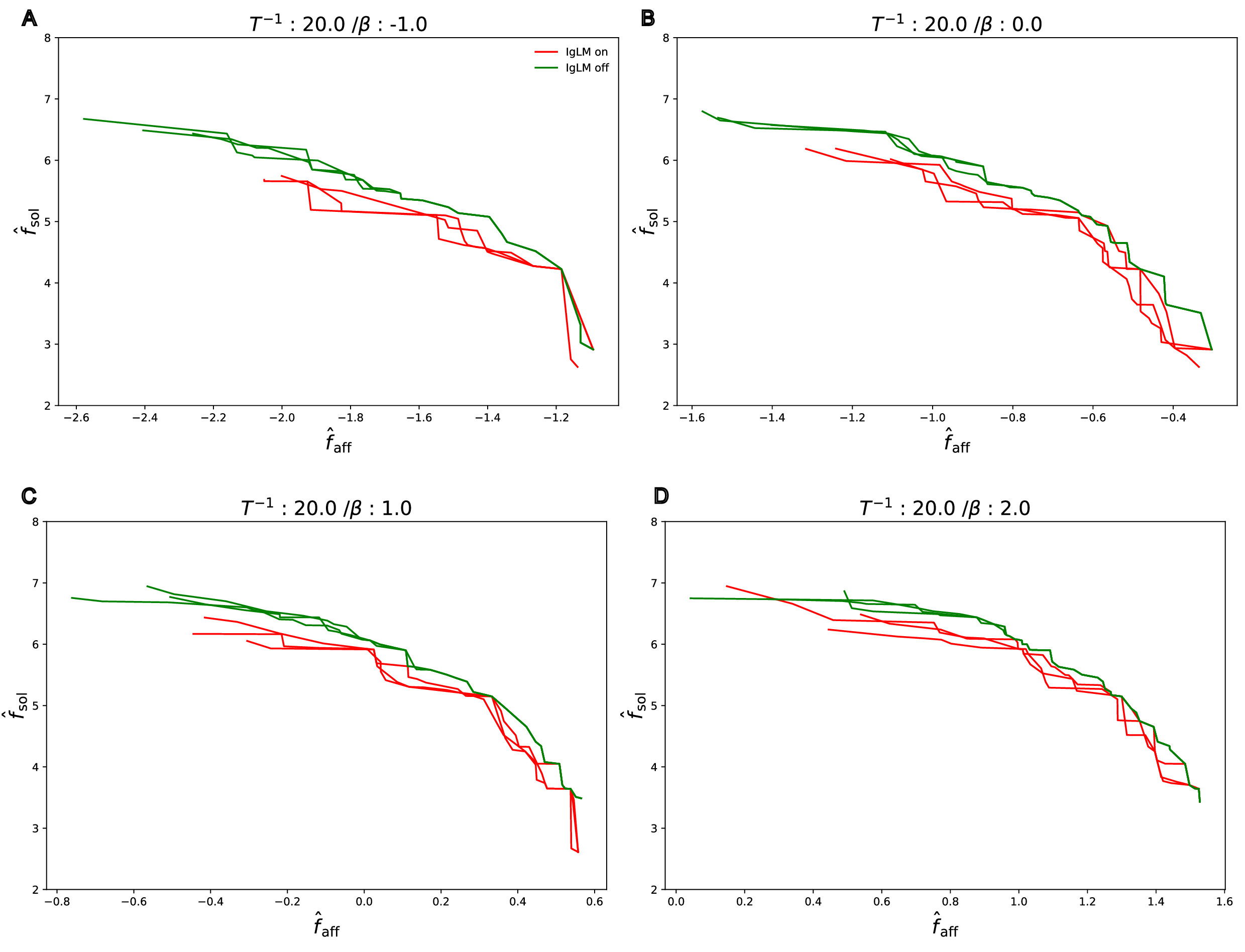

Supplement: figureS16.jpg [file KMAB_A_2584935_SM5420.jpg]

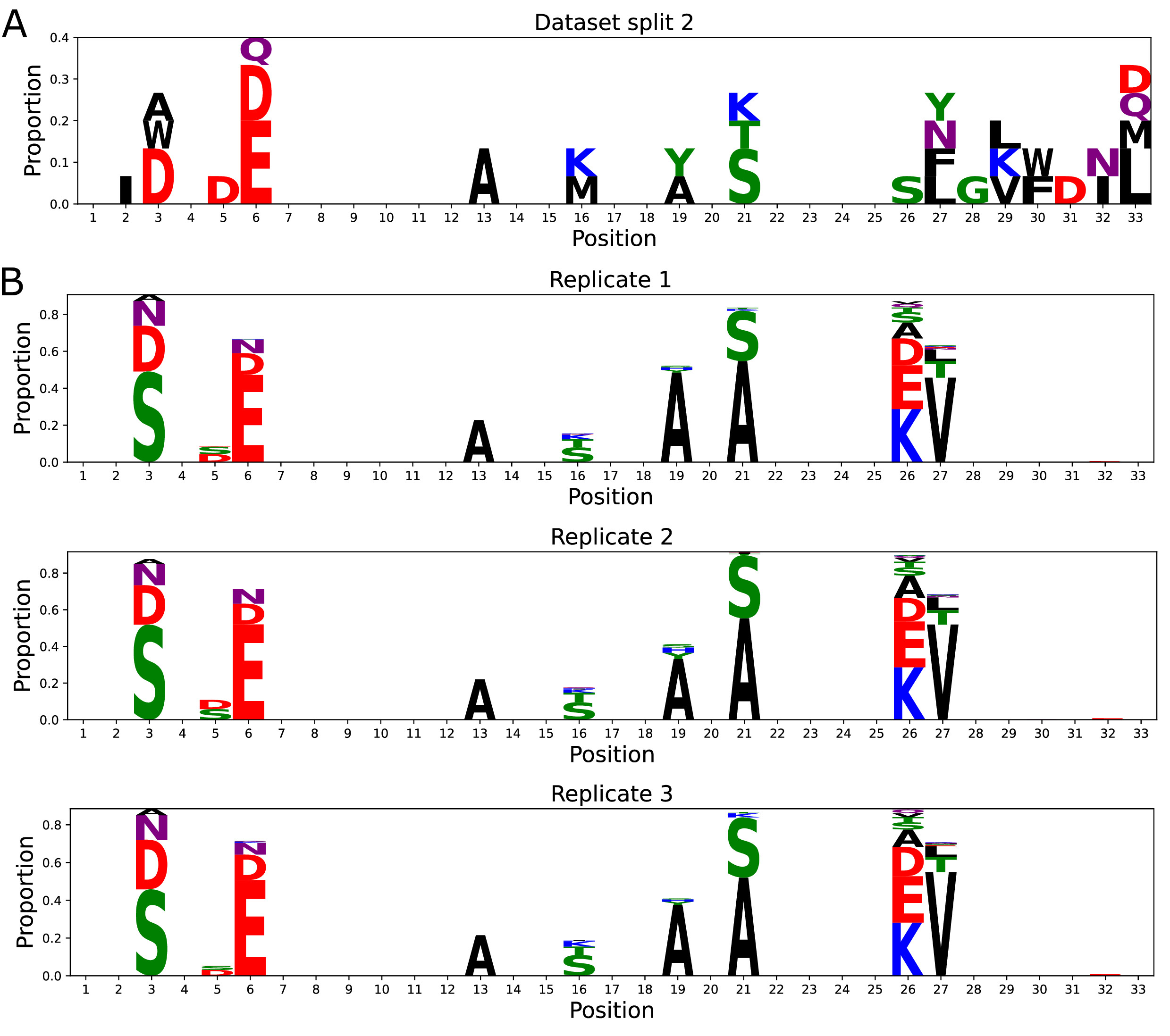

Supplement: figureS7.jpg [file KMAB_A_2584935_SM5419.jpg]

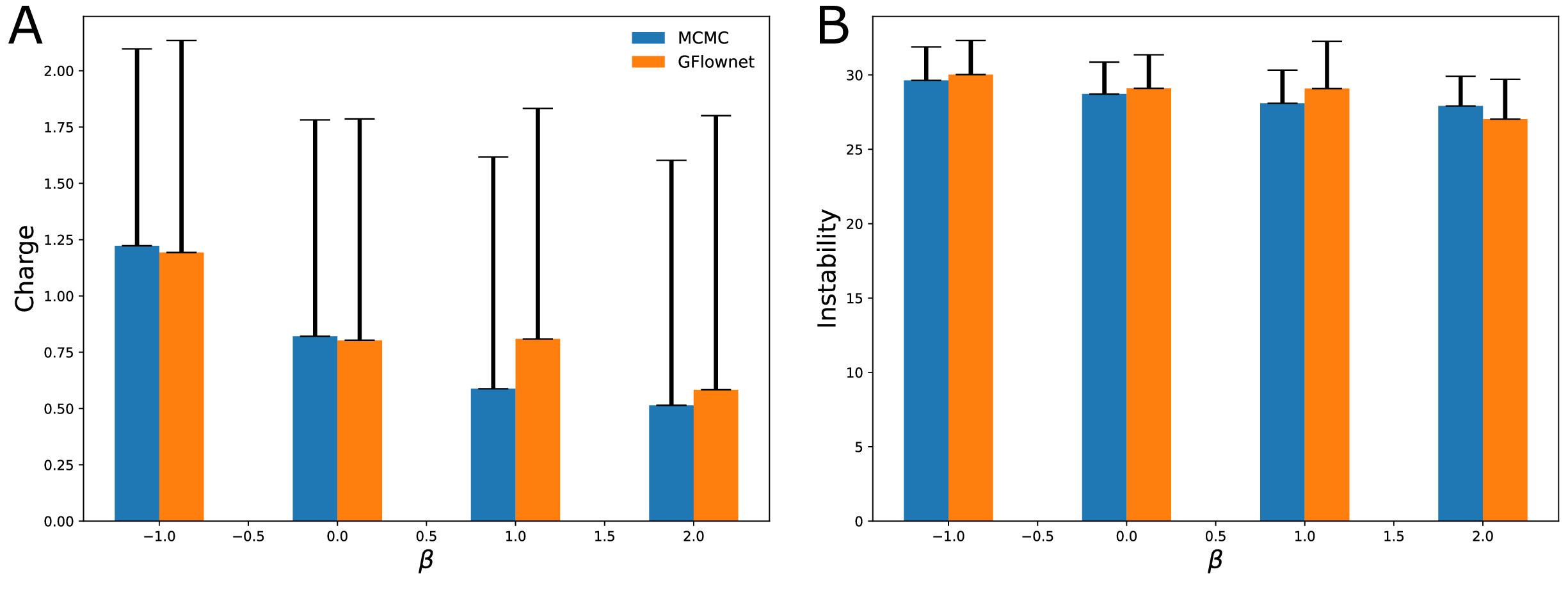

Supplement: figureS3.jpg [file KMAB_A_2584935_SM5418.jpg]

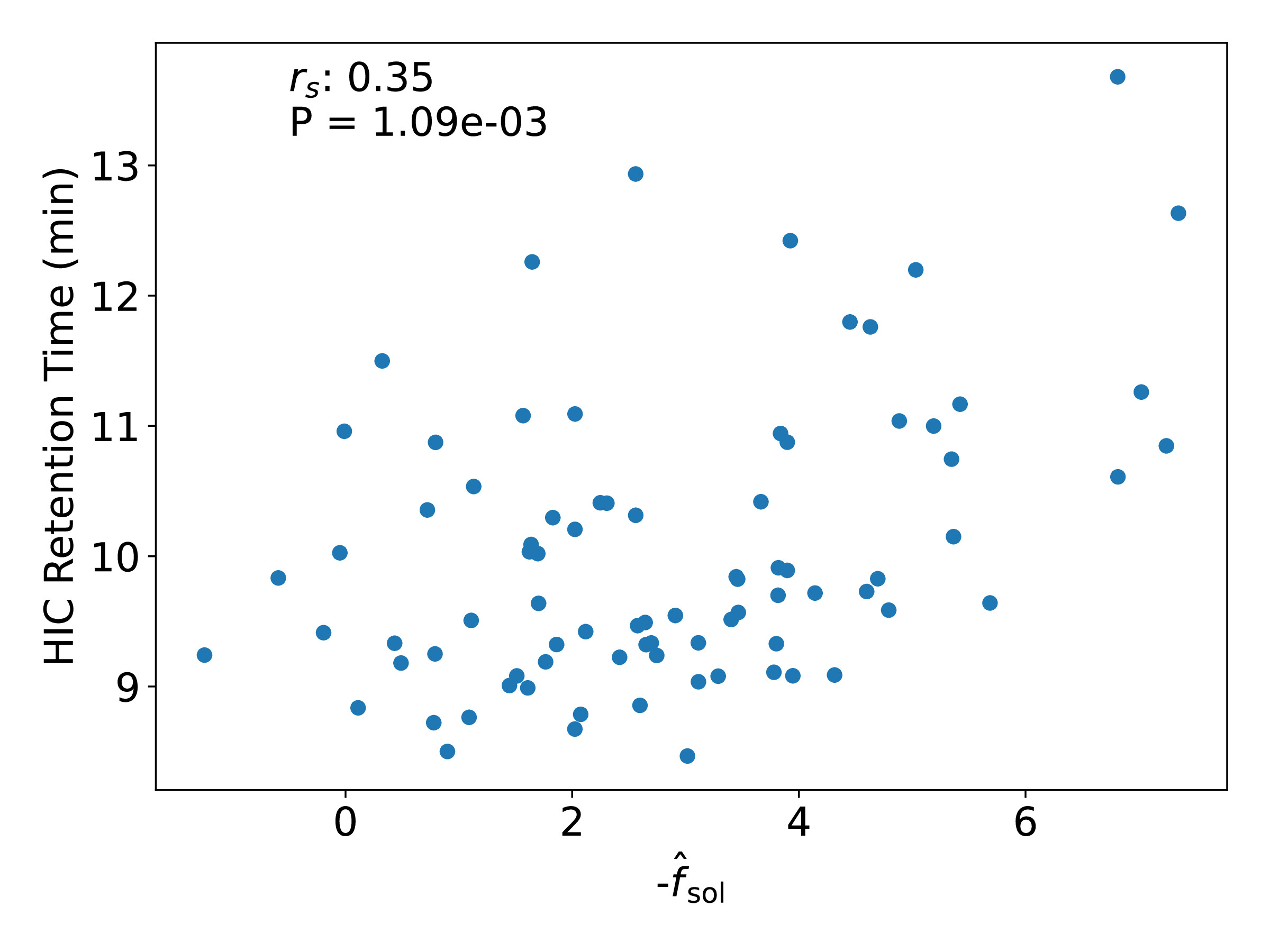

Supplement: figureS18.jpg [file KMAB_A_2584935_SM5417.jpg]
